# A twofold perspective on the quality of research publications: The use of ICTs and research activity models

**DOI:** 10.1371/journal.pone.0308952

**Published:** 2025-01-14

**Authors:** Jolanta Wartini-Twardowska, Natalia Paulina Twardowska

**Affiliations:** 1 Department of Business Informatics and International Accounting, University of Economics in Katowice, Katowice, Poland; 2 Department of Structural and Molecular Biology, University College London, London, United Kingdom; Kodolanyi Janos University of Applied Sciences: Kodolanyi Janos Egyetem, HUNGARY

## Abstract

Previous studies have highlighted the inherent subjectivity, complexity, and challenges associated with research quality leading to fragmented findings. We identified determinants of research publication quality in terms of research activities and the use of information and communication technologies by employing an interdisciplinary approach. We conducted web-based surveys among academic scientists and applied machine learning techniques to model behaviors during and after the COVID-19 pandemic. Using model-agnostic explanations, we identified the determinants of research publication quality across 66 activity models. These models reflect the variety of behaviors among academic scientists during and after the COVID-19 pandemic. Our two-fold perspective distinguishes between research activities of academic scientists who increase research publication quality and those who maintain it. Notably, our findings reveal a diversity within activity models in shaping research publication quality. Academic institutions can apply our approach to analyze research staff behavior, stimulate activities, and ensure alignment with institutional objectives, thereby fostering individual and team complementarity.

## Introduction

The quality of research and publications in academia significantly impacts scientists’ careers influencing their ability to secure grants, conduct further research, and advance in their work [cf. 1]. Recently, research quality has gained considerable attention, with studies examining the combined effects of research quantity and quality on economic growth [2]. Similarly, issues of questionable research practices and misconduct have been discussed [3], along with the impact of academic publication quality on launching public offerings [4]. Furthermore, research productivity, quality, and impact metrics have been compared [5], and open science practices have been recommended to increase public confidence in scientific findings [6]. Finally, an ongoing debate persists regarding different peer review methods and their influence on the quality of published articles [7].

When evaluating academic publications, it is essential to recognize high-quality research published in reputable journals, particularly those with a significant impact factor. Journals play a crucial role in curating and disseminating scholarly work by selecting and publishing the most impactful and relevant studies, thereby creating a platform for scholars to communicate their findings [8]. However, evaluating research quality remains highly subjective and challenging [9,10]. Prevailing assessment practices often place disproportionate emphasis on metrics such as the Journal Impact Factor. In response, initiatives such as the San Francisco Declaration on Research Assessment [10], the Leiden Manifesto with its ten principles for responsible research evaluation [11], and HuMetricsHSS [12] advocate moving away from these metrics. These initiatives address challenges such as ambiguous definitions and the inherent difficulties involved in assessing research quality.

Faculty members perceive quality subjectively due to the lack of standard definitions [9]. Moreover, universities increasingly prioritize the number of publications over their intellectual merit, as evidenced by faculty recruiting committees, promotions, and periodic evaluations [13]. Although some countries have implemented funding systems that tie rewards to the Journal Impact Factor (JIF), others have introduced”cash-for-publication” schemes to incentivize academic scientists to publish more. Consequently, this has led some academics to prefer less innovative but faster publication routes that can be easily monetized, occasionally resulting in falsified research [14]. Some countries, such as China, have banned cash rewards for publishing articles in indexed journals, opting instead to move towards “a balanced combination of qualitative and quantitative research evaluation” [15, p. 5]. Nevertheless, financial incentivization of academic publishing remains widely practiced globally.

In the “society of knowledge,” the research conducted by academic scientists is characterized by distinct action structures, meanings, and social or political dimensions. The research activities of academic scientists are expected to add value. Some scholars consider high-quality publications to be one of the cornerstones of the philosophy of science, alongside intervention (e.g., the number of scientific publications) and visibility (e.g., citation metrics) [16]. However, scientists who engage in falsification, misrepresentation, and ethical or methodological misconduct undermine trust in research. To combat these issues, fostering an open science culture—characterized by data sharing, preregistration of study protocols, and open access publishing—is a strategy for mitigating such practices [17].

The COVID-19 pandemic had substantial impact on universities, compelling them to rapidly adopt digital technologies. Although the pandemic’s impact on the use of information and communication technologies (ICTs) in basic or applied research has varied [18], digital and organizational changes are expected to persist beyond the crisis [19]. The benefits and challenges of utilizing ICT in education and academic collaboration have been widely reported [20]. However, more research is needed to explore how academic scientists have adapted to the changing conditions of their research work, the role of ICTs in this adaptation, and importantly, and its relationship with research publication quality. Additionally, investigating whether the pandemic has led to increased diversity in research activity models and whether gender plays a role remains crucial. Our definition of diversity encompasses differences in gender, age, academic position, science discipline, research type, and technology use [cf. 21,22]. We conducted a systematic literature review to address this knowledge gap. The review found that research activities among academic scientists have been examined in fragmented ways, focusing on types of research, popularization of research, collaborations, and interventions (e.g., publication counts), but lacked a comprehensive analysis. Furthermore, academic scientists are recognized as a heterogenous group, each with distinct ways of achieving their scientific objectives [23]. One reason for this diversity is the rapidly evolving work environment. Additionally, academic scientists’ information-seeking behaviors are undergoing significant changes [24].

Our study contributes significantly to research publication quality by providing an interdisciplinary approach to identifying diverse research activity models and their determinants. By leveraging activity theory as a theoretical framework for identifying behavioral models and offering suggestions for their re-evaluation, we uncover the diversity of activity models. Building on the works of Follett [25,26] and Shen et al. [21], our investigation assesses the impact of the pandemic on the diversity of research activities, ICT use, and their relationships with research publication quality.

Our approach involves machine learning techniques and model-agnostic explanation method, enabling us to explore differences in individual or group behavior. Our findings indicate that publication quality can be boosted by selecting appropriate activity models that incorporate diverse determinants of research activities and ICTs.

Consequently, we propose a new approach to identify individual or group differences and boost research publication quality by integrating different models. By adopting an interdisciplinary approach, we highlight opportunities to enhance quality by acknowledging synergies between research activity model synergies.

The remainder of this paper is structured as follows: First, we present a tabulated synthesis and co-occurrence network of terms from a systematic literature review. In the next section, we develop research questions and hypotheses. We then describe the methods and techniques used in this study. This is followed by the results, implications for academic scientists and university authorities, and discussion. Finally, we conclude the paper by addressing limitations and future research directions.

## Theoretical background

We drew the theoretical background for this study from a systematic review of previous research from 2020 to March 2023.

### The systematic review process from 2020 to March 2023

We followed a sequential process for our systematic review, as shown in Table 1. This involved selecting the appropriate database, criteria, and terms, as well as conducting a narrative synthesis. The elements of a systematic review are widely agreed upon, and we integrated them into our review based on previous research [27,28]. Due to the lack of consensus in the literature on specific criteria for excluding studies from the qualitative synthesis (e.g., methodological limitations [29]), we established clear and defined inclusion criteria (see Table 1) to assess the quality of the published articles. Records were downloaded from the Web of Science Core Collection database on March 17, yielding 597 articles (S1 Table can be found at [30]). Among these, only 34 qualified for the quantitative synthesis. Our objective was to report the key insights and characteristics of the studies included in this systematic review (see Appendix 1 in S1 Appendix).

**Table 1:** Flow diagram for the systematic literature review process.

Searched database		The Web of Science Core Collection provided by Clarivate Analytics	
			
Research questions		RQ1 and RQ2 listed in part Hypotheses Development	
			
Inclusion criteria for the review		Articles; commentary, review articles (including early access) for the period 2020 to March 2023; all categories; all journals	
			
Screened terms		Two searching: (quality AND academic AND research AND publications) NOT (education OR grants OR students OR control quality OR services OR information OR instruction OR information literacy). (quality AND academic AND publications AND collaboration AND technology) NOT (education OR children OR patients OR schools OR students OR city OR adults OR citizen)	
			
Screened records		Total records screened in the review(n = 597)	
			
Exclusion criteria for the review and excluded records		Books or non-English articles from medical and health sciences, articles unrelated to research questions; articles without methodology or with inconsistent methodology; published in journals with Impact Factor under 17 or under Q3, only available for a fee or from “predatory” journals. Total records excluded from the review (n = 563)	
			
Included records		Total records included in the review (full texts) (n = 34)	
			
Data synthesis		Quantitative synthesis of results	

Note: RQ—the research question of this study.

Source: Based on the PRISMA guidelines [28].

### Exploring relationships using text mining

We used the open-source VOSviewer software (version 1.6.19) to analyze content and visualize the co-occurrence network of important terms extracted from the articles [31]. Specifically, we examined terms from abstracts and keywords, focusing on the co-occurrence relationships between them [32]. Standard parameters, including the full counting method, were applied. Although thresholds may vary, the minimum threshold for keyword co-occurrences was set to four, following Guleria and Kaur’s work [33]. VOSviewer identified 69% of the most relevant terms.

**Fig 1:**
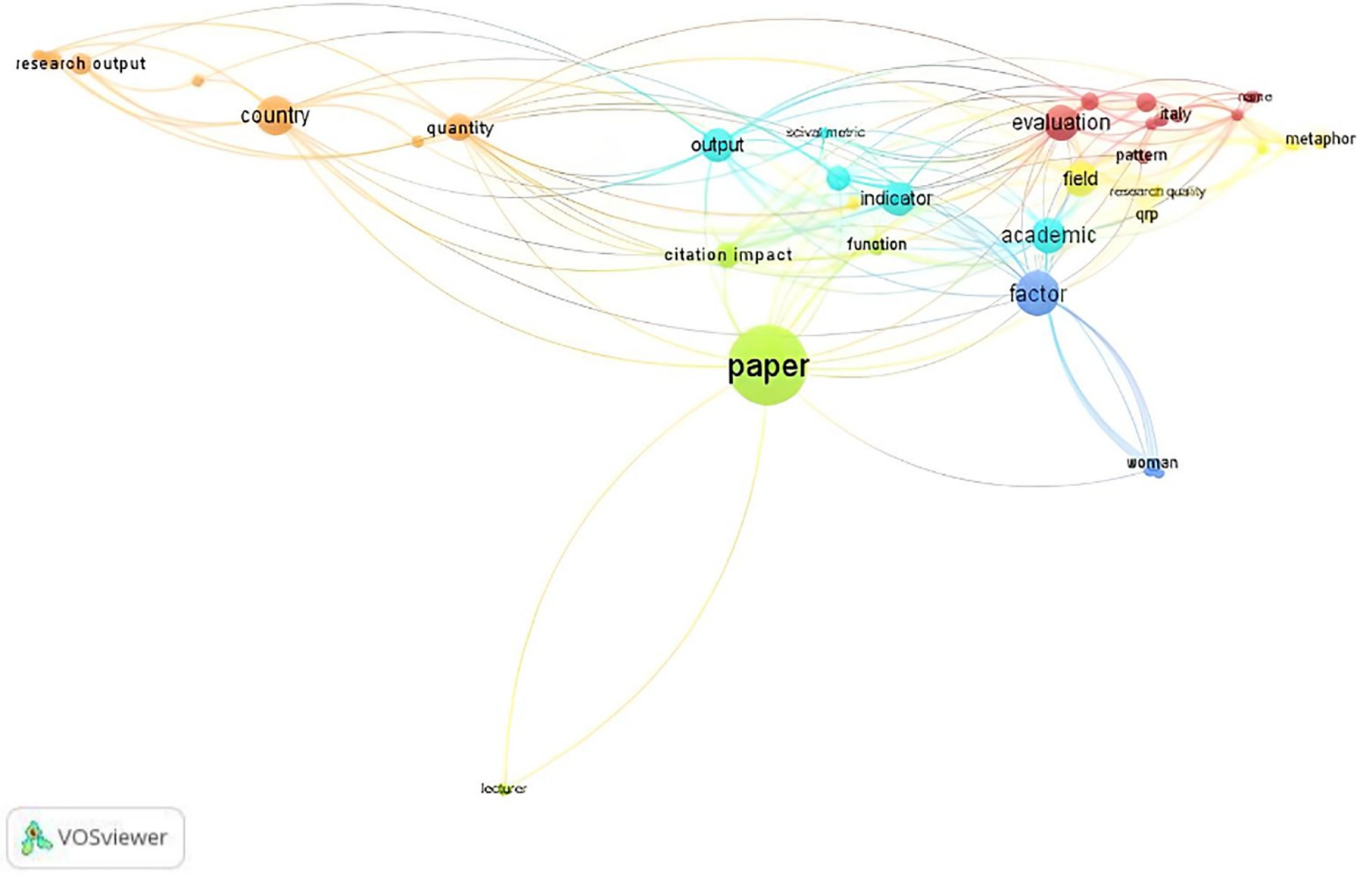
Co-occurrence network map of the items related to research publication quality.

We filtered out terms deemed insignificant, such as including “question,” “use,” “part,” “year,” “period,” “extent,” “data,” “importance,”“ analysis,” and “article,” as they did not contribute to our study on the quality of research publication and the exploration of relationships between key terms. Additionally, we excluded the term “article” due to its similarity to “paper,” which is more frequently used in abstracts. Notably, excluding “article” did not alter the number of clusters, as shown in Fig 1. We also employed association strength as a normalization method, establishing a minimum cluster size of two and merging small clusters. Finally, we visualized a map of the co-occurrence network of terms extracted from the systematic literature review.

VOSviewer identified 38 significant terms based on the quantitative analysis of the 34 articles listed below in alphabetical order (Table 2).

**Table 2:** Significant terms from the systematic review related to activities affecting the quality of research publications.

Significant terms		
“academic research”	“italy”	“qrp”
“academic”	“journal ranking”	“quantity”
”career progression”	“lecturer”	“research area”
”citation impact”	“metaphor”	“research assessment”
“country”	“metric”	“research collaboration”
”economic growth”	“name”	“research output”
“environmental accounting”	“new bottom line”	“research quality”
“evaluation”	“output”	“scival metric”
“factor”	“pakistan”	“scopus”
“field”	“paper”	“sII”
“function”	“pattern”	“ukrainian economic research”
“gender”	“professor”	“woman”
“indicator”	“promotion”	

We constructed a co-occurrence network map of the important items, which were classified into six clusters using VOSviewer (Fig 1). Each assigned item is supported by the strength of the links between the items. The items with the fewest links were excluded from the map. In Fig 1, the size of an item circle is determined by its weight [32].

Cluster #1–Red

The largest red cluster comprises ten items. Notably, “evaluation” is rooted in subjective interpretations of research quality and prestigious journals [2], and it stands out most prominently on the map (Fig 1). Other significant terms include (ii) “research assessment,” contextualized within the evaluation of Italian universities and research institutes or the effectiveness of policy [34,35]; (iii) “scopus” as a data source for research assessment [36,37]; (iv) “pattern” of international co-authorship [38]; (v) “journal ranking” based on an integer Data Envelopment Analysis (DEA) model for evaluating academic journals [39]; (vi) “Italy” reflecting Italian strategies for publication [35,40]; (vii) dishonestly added “name” of not-involved authors to articles [41] for (viii) “promotion” to create increased demand for publications [13,35]; (ix) calculation of “research collaboration” as presented by Sarkar et al. [42] using the Constant Elasticity of Substitution function; and finally, “environmental accounting” research published mainly in non-top-tier journals but holding a relatively strong position [43].

Cluster #2–Orange

The orange cluster, although smaller (seven items), prominently emphasizes the aspect of “quantity” [2,8] in research considered for scientific evaluation [44]. Key items within this cluster include: (ii) identifying similarities and differences by “country” to choose the right journal, where the quality of a journal is measured based on prestige, impact factor, and indexation in the WoS or Scopus databases [36]; (iii) measuring”research output” by the impact of citation numbers on economic growth [2]; (iv) recognizing that”economic growth” depends on improving the quality of research in the Middle East & North Africa (MENA) countries; (v) acknowledging a negative impact of quantity in almost all “research areas” on economic growth in MENA countries [2]; (vi) highlighting the term “pakistan” as a critical determinant of quantity contributing to highly cited Pakistani researchers [34]; (vii) noting that “ukrainian economic researcher” has a comparably small share of co-authored publications [37].

Cluster #3–Yellow

As for the yellow cluster, it contains seven items related to the recognition of high-quality research. These include: (i) the “field” of study; (ii) the use of metrics such as the superior identification index to evaluate the capability of academic journals [8]; (iii) the influence of questionable research practices “qrp” on research credibility [3]; (iv) the “research quality” of PhD students’ publications, shaped by pressures to meet quantified output standards, limited publication options, and unethical practices such as paper brokering and purchased authorship [45], as well as the positive and robust correlation between high-quality journal publications in early career stages and long-term scientific success [46], and the establishment of rigorous reviews of high-quality pull requests for open-source research software to enhance the quality of scientific research and foster the development of advanced algorithms within community software ecosystems [47]; (v) the increased pressure to publish in A-list journals that value “academic research” [13]; (vi) the metaphor of the publication game and its unforeseen or undesirable impact on academic behavior [48]; (vii) the “new bottom line,” encapsulated in the phrase “a win is a win,” emphasizing the value of any published research [13]; (viii) the reduction of academic isolation and improvement of research quality through increased collaboration between researchers from the North and the South [49].

Cluster #4–Green

In the green cluster (containing five items, Fig 1), (i) “paper” is the most prominent item among all clusters. Subsequently, (ii) the “citation impact” of the paper did not increase, although the paper received subsidies [14]. Articles by repeat authors showed a decline in citation impact with each additional repeat authorship [50]. The third item is the proposal of a scoring method for smaller universities using the “function” of global influence in terms of citations [42], followed by (iv) “lecturer” and (v) “professor” who should be penalized for following bad practices and publishing articles in predatory journals [51].

Cluster #5–Turquoise

In the turquoise cluster, (i) “academic” has the strongest total link strength. Next, (ii) research “output” is visualized on the map; (iii) “number” [5], and (iv) “metric” are linked to the number of outputs and citations, which increased with academic level and career stage; (v) “scival metric” was used for the evaluation of “academics” performances [5]; (vi) “indicator” was utilized for the measurement of publication impact [52; and (vii) “scientific development” in terms of its primary indicators [14], such as the number of articles published in high-impact scholarly journals and journal ranking [39].

Cluster #6–Blue

The last cluster contains four items: (i) “factor,” which involved contributions to citation success [38] or the evolution of the researcher community [53]; (ii) “women,” who paid more attention to publishing high-quality articles compared to male researchers [54]; (iii) the impact of conference presentations on career progression, particularly the positive link between conference presentations and publishing in high-quality journals [55]; and (iv) “gender,” specifically its impact on journal selection [54].

According to the map shown in Fig 1, five primary terms were used most frequently across all clusters. These terms were ranked in descending order of strength. The first term, “paper,” appears in 18 links and has a total link strength of 297, representing 21% of the total link strength across all clusters. The second term, “academic,” is used in 20 links with a total link strength of 197, accounting for over 15% of the overall link strength. The third term, “quantity,” is included in 18 links and has a total link strength of 166, representing nearly 13% of the total link strength of all the clusters. The fourth term, “output,” appears in 17 links and has a total link strength of 158, representing over 12% of the total link strength across all clusters. Lastly, the fifth term, “metric,” is used in 17 links with a total link strength of 139, accounting for approximately 10.8% of the overall link strength.

The map indicates that most terms combined with “research quality” are visually prominent, except for “quantity.” However, strengthening these relationships is necessary. This enhancement is critical for shedding light on whether the behaviors of academic scientists who focus on increasing quantity differ from those who maintain the quality of their research publications. In addition, the survey points to a gap in research analyzing models of ICT use in academic research that remain underrepresented on the map. Therefore, we address this gap by examining the different roles of ICT in models of research activities.

## Hypotheses development

This study focuses on academic scientists who fall within Stoke’s four-quadrant research model [56]. These are scientists engaged in basic and/or applied research or those who do not conduct any research. Most respondents who participated in the study were engaged in basic and applied research during Periods 1 and 2.

Academic scientists undergo periodic evaluations that rely on quantitative measurements and incentives for immediate knowledge production [44]. Indicators such as the number of scientific publications, citations, and international collaboration are crucial for measuring efficiency. For instance, the number of scientific publications plays an important role in efficiency (e.g., in Iran in the human sciences, social sciences, and natural sciences), whereas international collaboration ranks second in the social sciences [57].

However, citations, while widely used, cannot serve as unbiased proxies of the scientific quality of publications, as they reflect a combination of intellectual achievement, social relationships, and ideological factors [58]. This highlights the need for more sophisticated analytical approaches to evaluate research quality. Tools such as the Scopus database, which provides metadata and comprehensive author and institutional profiles, enable advanced analyses, including researcher mobility and spatial bibliometrics, although these capabilities have not yet been explored in the context of differences in publication quality during and after the pandemic [59].

Academic leaders improve their research productivity when they are confident in the high quality of their research [60]. Our study addresses a new aspect of the quantity of scientists’ publications and uncovers models of research activities in which maintaining or increasing the number of publications has a differential impact on quality during or after the pandemic.

While some hold the view that international collaboration, with expectations of increased research quality, greater output, and higher potential, drives academic scientists to build international reputations [60,61], it can also create tensions, especially in partnerships, when a collaborator demonstrates clear dominance [62]. Under these conditions, collaboration may not yield satisfactory results [61].

However, solo-authored work in the early careers of academics has become increasingly rare. This alarming regularity persists among economists with different impact profiles, irrespective of productivity [63]. Academics manage team formation and engage in positive assortative matching, and “[…] in all fields of science and patenting, team impact is weighted toward the lower-impact team members […] with implications for the output of specific teams […]” [64 p. 13885].

We present new evidence based on the dual perspective (of increasing or maintaining quality) and assert that most types of collaboration do not occur in both groups of academic scientists, thereby emphasizing differences observed during and after the pandemic.

Based on Engeström’s work [65] and the suggestions of Bence and Oppeheim [66] regarding evaluation criteria for academic scientists, we selected some of these criteria as variables influencing the quality of research publications (Table 3).

**Table 3:** List of variables and their descriptions used in the study on research publication quality during and after the pandemic.

Abbreviation of variable	Name of variable	Description of variable	Description imposed by the 5-point Likert scale/ dichotomized scale	
Qa (Surveys 1 and 2)	The quality of research publications	The change in the quality of research publications	Definitely smaller 1 Rather smaller 2 Neither smaller nor bigger 3 Rather bigger 4 Definitely bigger 5	/ 0 / 0 / 0 / 1 / 1
Qn (Surveys 1 and 2)	The quantity of research publications	The change in the number of research publications		
BR (Surveys 1 and 2)	Basic research	Carrying out research focused on basic research	Never 1 Seldom 2 Sometimes 3 Often 4 Very Often 5	
AR (Surveys 1 and 2)	Applied research	Carrying out research focused on applied research		
Research (Surveys 1 and 2)	Research	The place of research among other academic scientists’ activities	Rank 1 (-First place) Rank 2 (Second place) Rank 3 (Third place) Rank 4 (Last place)	
Grant_wr (Surveys 1 and 2)	Grant writing	The place of grant writing among other academic scientists’ activities		
Com_apps (Surveys 1 and 2)	Communication apps	Communication apps used in research (e.g., Skype, WhatsApp, Google Meet, Messenger, MS Teams)	Never 1 Seldom 2 Sometimes 3 Often 4 Very Often 5	
E_learn_platf (Surveys 1 and 2)	E-learning platforms	E-learning platforms used in research (e.g., Moodle, Google Classroom, Zoom, Docebo, Wiz IQ, ATutor, MS Teams)		
Online_conf (Surveys 1 and 2)	Online conferences	Attendance at online conferences, webinars, etc.	Definitely unimportant 1 Rather unimportant 2 Neither important nor unimportant 3 Rather important 4 Definitely important 5	
Trad_conf (Surveys 1 and 2)	Traditional conferences, seminars	Attendance at traditional conferences, seminars, etc.		
Social_media (Surveys 1 and 2)	Social media	Social media used in research (e.g., Facebook, Twitter, LinkedIn, YouTube, Instagram, blog sites)	Never 1 Seldom 2 Sometimes 3 Often 4 Very Often 5	
Stat_softw (Survey 1)	Statistical analysis software	Statistical analysis software used in research (e.g., MATLAB, Statistica, SPSS, SageMath)		
Qu_softw (Survey 1)	Questionnaire software	Questionnaire software used in research (e.g., Candel, Charted, Datawrapper, Leaflet, LimeSurvey, SurveyMonkey)		
Online_db (Survey 1)	Online databases	Online databases used in research		
E_journ (Survey 1)	E-journals	E-journals used in research		
Print_journ (Survey 1)	Print journals	Print journals used in research		
E_book (Survey 1)	E-books	E-books used in research		
Print_book (Survey 1)	Print books	Print books used in research		
Collab(foreign_r) (Survey 1)	Collaboration with foreign academic scientists	The importance of collaboration with foreign scientists in research	Definitely unimportant 1 Rather unimportant 2 Neither important nor unimportant 3 Rather important 4 Definitely important 5	
Collab(uni_faculty) (Survey 1)	Collaboration with your university or faculty’s academic scientists	The importance of collaboration with academic scientists in research at the respondent’s university		
Collab(postdoc_st) (Survey 1)	Collaboration with post-docs, grad students	The importance of collaboration with post-docs and grad students in research at the respondent’s university		
Collab(r_outside_uni) (Survey 1)	Collaboration with domestic academic scientists from outside your university	The importance of collaboration with domestic scientists in research from outside the respondent’s university		
Collab(w_others) (Survey 1)	Collaboration with others	The importance of collaboration with non-scientists in research		
Age (Surveys 1 and 2)	Age		< 20 20–34 35–49 50–68 >69	1 2 3 4 5
Gender (Surveys 1 and 2)	Gender		Unspecified 0 Female 1 Male 2	
PL_Abroad (Surveys 1 and 2)	Country of work		Poland 1 Abroad 2	
OECD (Surveys 1 and 2)	Science discipline best describing research		Social sciences 1 Engineering and technology 2 Humanities 3	Natural sciences 4 Medical and health sciences 5 Agricultural sciences 6
Scientific_position (Surveys 1 and 2)	Current scientific position		Ph.D.-student 1 Lecturer 2 Assistant Professor 3 Associate Professor 4 Professor 5	Retired 6 Researcher 7 Assistant 8 Other 9
Teaching_model (Surveys 1 and 2)	Teaching model best describing university		Traditional 1 Traditional and online 2 Online 3	

Source: Adapted from [67].

According to Shen et al. [21], many organizations fail to account for individual differences when making and implementing performance appraisals. This oversight often leads to undervaluation of diversity, which is crucial for the success of any organization. The first step in managing diversity is to measure it. Moreover, the lack of a proper measurement system indicates that universities and other institutions are cannot effectively leverage the diversity in their staff. Thus, this study formulates the following general question and the corresponding hypotheses (H1a to H1f). All hypotheses were tested irrespective of gender and across each analyzed period.

RQ1 (Surveys 1–2): Do the research activities undertaken by quality-increasing academic scientists differ from those undertaken by quality-maintaining academic scientists?

H1a (Surveys 1–2): Increasing the number of scientific publications is important for increasing or maintaining research publication quality.

H1b (Surveys 1–2): Prioritizing research is important for increasing or maintaining research publication quality.

H1c (Surveys 1–2): Prioritizing grant writing is important for increasing or maintaining research publication quality.

H1d (Survey 2): Collaboration with foreign academic scientists is important for increasing or maintaining research publication quality.

H1e (Survey 2): Collaboration with domestic academic scientists is important for increasing or maintaining research publication quality.

H1f (Survey 2): Collaboration with others (e.g., business professionals) is important for increasing or maintaining research publication quality.

Previous studies have revealed that ICTs transform the academic landscape and reshape the future of university innovations, shedding light on the following:

- What is the impact of ICT on research work, and what are the most commonly utilized ICTs? Which ICTs facilitate researcher collaboration or idea sharing? [18,68,69].
- How does ICT significantly enhance scientists’ performance or productivity? [70–72].
- What are academics’ attitudes towards ICTs in (doctoral) research processes? [73,74].
- What are the barriers to implementing ICTs in an academic environment? How do the complex interplay between policy and infrastructural challenges that influence ICT implementation? [75,76].
- What are the benefits of digital technology in learning? [77,78].
- How can ICTs be used for research, scientific dissemination, and data analysis? Which software tools enable academic scientists to efficiently manage large datasets, perform complex statistical analyses, and visualize data to enhance the credibility of research findings? [39,79,80].
- What are the aspects of ICT (innovation) adoption in Library and Information Science? How does ICT support managing or providing access to electronic information, and does ICT access allow for comprehensive literature reviews and staying updated with the latest research trends? [81,82].
- How can ICT improve the peer review process? What is the value of plagiarism detection software in evaluating cross-disciplinary articles? [83,84].

Based on the evidence gathered, our study is thoroughly justified in focusing on unexplored determinants that contribute to academic success and maintain or increase the quality of research publications. Specifically, we aimed to investigate how research publication quality (maintained or increased) is impacted by ICTs, depending on the gender of the academic scientists and the analyzed periods (during and after the pandemic).

The digital age has led to a growing prevalence of e-publications, and media experts predict completely replacing print publications with digital publishing [85]. In this study, we examined whether non-digital or digital media are crucial for academic scientists to increase or maintain the quality of research publications, including whether digital media have fully replaced print books. Gunapala et al. [82] reported that printed materials were rarely used in Australian university libraries. In Ghana, access to information resources in both print and electronic formats has increased [86].

Our study also fills a research gap regarding the use of ICTs to increase or maintain the quality of research publications. Although a near-moderate correlation was observed between the use of social media and applied research during and after the pandemic, the correlation between using the aforementioned digital technologies and basic research was the weakest [18]. According to Ziemba and Eisenbardt [19], the COVID-19 pandemic has accelerated the use of ICTs to support research, and this change has continued after the pandemic. The adoption of alternative ways to communicate science, including online seminars [87] or virtual conferences with live videos and chats [88], could be helpful for academic relationships, particularly during disruptive periods (e.g., during a pandemic). Therefore, it would be interesting to investigate the ICTs used in university research to assess the relationships between ICT use and the increase or maintenance of research publication quality. Following RQ1, we formulated the next research question (RQ2) along with three corresponding hypotheses (H2a, H2b, and H2c):

RQ2 (Surveys 1–2): Do quality-increasing and quality-maintaining academic scientists differ in their use of ICT in research?

H2a (Surveys 1–2): The frequent use of (digital) communication technologies in research is important for increasing or maintaining research publication quality.

H2b (Survey 2): The frequent use of (digital) information technologies in research is important for increasing or maintaining research publication quality.

H2c (Survey 2): The frequent use of non-digital information technologies in research is important for increasing or maintaining research publication quality.

We examined the hypothesized importance of each variable for research publication quality across both analyzed periods. Differences in their effects on research publication quality were identified and thoroughly analyzed using the developed models.

## The framework of the activity system

Based on the activity theory, we established a general framework for our study. Initially developed by Vygotsky in the 1920s and the early 1930s, the activity theory was further refined by Leontiev and Luria, who introduced the concept of “activity” [89]. Engström’s structure of activities in this study became a starting point for exploring academic scientists’ behavior [65].

Activity theory provides a lens to understand how individuals interact in their research environments. Although it does not prescribe specific research methods or techniques, Leontiev’s concept of human activity defines relationships among the “subject-object-community.” These relationships extend to include “instruments, rules, and division of labor” [65].

This activity can be understood through tools (in our study, ICTs) that mediate between academic scientists and the object of the activity (e.g., research).”All human experience is shaped by the tools […]" [90, p. 10]. The relationship between subject and community is mediated by rules, and the relationship between object and community is mediated by the division of labor. Technology is a crucial enabler for activity. ICT facilitates activity by linking academic scientists [cf. 89]. The collaborative nature of research activity is crucial. Although academic scientists can act individually within organized activity systems such as universities, their ability to act is based on forming communities and collaborating with each other [cf. 65].

Activity theory provides our research with a basic theoretical framework for selecting variables for activity models based on nodes, such as objects (i.e., basic and applied research), subjects (i.e., academic scientists), tools (i.e., ICT), and communities (i.e., collaboration). Because we do not focus on the interrelations between activity nodes and outcomes, activity theory remains fundamental for interpreting the results of our comprehensive data analysis. It allows us to identify activity models across the two periods, including changes in ICTs used to increase or maintain publication quality.

Academics are autonomous and influential individuals who control their behaviors, whereas universities often impose constraints and shape their activities. We analyzed activities using Poole and Van de Ven’s dimensions [91], focusing on micro-level (universities) and individuals’ activities (academic scientists’ activities). Although universities share common objectives, particularly in relation to shared values such as research publication quality, we investigated the activities and ICT tools crucial for increasing or maintaining research publication quality.

## Methods

### Study design

This study employed a survey aligned with an interactive approach to Internet-mediated research as the primary method. Web surveys yield results with comparable validity to those obtained using traditional methods [92]. A computer-assisted web interview method (CAWI) was used to recruit respondents and collect data. Online questionnaires (Surveys 1 and 2) were designed using LimeSurvey, through which respondents were recruited.

This study employed two non-probabilistic sampling techniques. The purposive sampling targeted academic scientists, focusing on a specific population to collect more usable questionnaires within a shorter time frame. Purposive sampling is suitable for both qualitative and quantitative research [cf. 93,94]. The second technique, self-selection, involved posting the online questionnaire on a specific website. Self-selection means that individuals are free to choose themselves for participation in the survey. As a result, only respondents with internet access who visited the website and decided to engage with the survey participated. In self-selection, scientists lack control over the selection process, often resulting in significant bias in the estimates. Furthermore, the accuracy of these estimates cannot be computed [95]. Nevertheless, non-probabilistic sampling did not undermine the validity of this study, as its primary aim was to assess whether two or more variables are related as theory anticipates, or to reject the null hypothesis that two variables are completely unrelated. In such cases, non-probabilistic samples are appropriate [96].

A general protocol for research participation was applied to the web surveys ensuring that they were anonymous and voluntary [97]. The survey consisted of two versions: the extended online questionnaire (Survey 1), and its shorter version (Survey 2).

All online questionnaires used a 5-point Likert scale, chosen for its ease of interpretation, and were prepared in English. The 29 questionnaire questions (QQ) included in Survey 1 and 17 in Survey 2 are listed in S2 Table [30].

In late May 2020, Survey 1 was pretested by 17 academics: ten from Poland, two from the United Kingdom, two from the United States, and one each from Germany, Nigeria, and Slovakia. While minor formal, technical, and linguistic changes were made, no content changes were required [cf. 18].

After collecting responses on the quality of research publications using a Likert scale, we dichotomized the responses [cf. 98] for model classification. The same technique was applied to the quantity of research publications.

### Data collection

To increase response rates, academic scientists encouraged their colleagues to participate, and the web survey was distributed via direct emails to respondents. Additionally, the project, along with a link to the extended questionnaire, was shared on the ResearchGate platform and Facebook fan pages. Frequent reminders were provided [99].

Two online questionnaires (Surveys 1 and 2) were administered from June 11, 2020, to August 18, 2020. This resulted in 347 responses for Survey 1 and 982 responses for Surveys 1 and 2.

This study used two independent samples from two periods (during and after the pandemic). Both web surveys differed in the number of responses and questions collected.

After screening and excluding incomplete responses, 152 complete responses were obtained for Survey 1 (Periods 1 and 2), 476 for Surveys 1 and 2 (Period 1), and 454 for Surveys 1 and 2 (Period 2). The datasets used in this study were organized into four sheets within a single Excel file, referred to as S3–S6 Tables in S3 Table in this article, and were uploaded to the Zenodo repository [67]. A summary of the survey demographics and descriptive statistics, can be found in Appendix 2 in S1 Appendix and S4 Table [30].

As a larger sample size yields richer information, reduces uncertainty, and enhances stability, we incorporated responses from Survey 2 into our analysis to explore whether analyzing the larger dataset with a smaller number of variables would lead to differences in activity models and ICT use between the two periods. We adopted a strategy of combining datasets from two surveys covering both periods, ensuring consistency in data format [cf. 100]. Moreover, incorporating data did not pose semantic challenges, as the precise meaning of questionnaire questions was ensured. We followed a stepwise procedure for analyzing the results: first examining the same questions from Surveys 1 and 2 for Periods 1 and 2, and then extending our analysis to Survey 1 for Periods 1 and 2.

### Data analysis

First, the data were stored in a single format, i.e., MS Excel.

Second, we analyzed the data using Python software (version 3.7.0).

Third, we employed the LazyPredict package for Python [101] to identify the appropriate classifiers for the four datasets: Surveys 1 and 2 for Periods 1 and 2. Out of 27 methods, we selected the five best-performing machine learning classifiers. To ensure reproducibility, we set specific values for the "RANDOM_STATE" parameter for each survey and period: 988 for Surveys 1 and 2, Period 1 (Y and N); 215 for Surveys 1 and 2, Period 2; 140 for Survey 1, Period 1 (Y and N); and 607 for Survey 1, Period 2 (Y and N).

For our imbalanced datasets, we followed standard steps to measure performance more precisely. These steps included defining a dataset, creating a pipeline, and evaluating the pipeline as outlined by Liu [102].

Defining a dataset: We applied a repeated stratified k-fold cross-validation, a widely used method, to estimate the performance and configuration of machine learning algorithms on the datasets (n_split = 10, n_repeats = 3). The repeated k-fold cross-validation enhanced the estimated performance of the machine learning model, yielding a more accurate estimate of the unknown underlying mean performance of the model. In total, k models were fitted and evaluated on k holdout test datasets, and their average performances were reported [cf. 103,104].

The scikit-learn “train_test_split” function splits our four datasets into training and testing sets. We allocated 80% of the observations to the training sets and the remaining 20% to the testing sets [102].

Creating a pipeline: The “imbPipeline” was configured as an estimator within GridSearchCV. All models were evaluated using SMOTE oversampling on imbalanced classification datasets [104]. Additionally, we performed preprocessing using RobustScaler to mitigate the effects of outliers [cf. 105].

The models were trained and evaluated using GridSearchCV [106]. Furthermore, GridSearchCV automated the identification of the best hyperparameter combinations. Our datasets are imbalanced because the classification categories are not equally represented. We applied SMOTE, a well-established over-sampling method for minority classes, to address the class imbalance problem [104,107]. Then, we mainly used standard parameter settings for the selected classification algorithms [cf. 106], specifically for “imbPipeline” and “param_grid” as detailed below. These standard settings are based on widely adopted practices in machine learning for handling imbalanced datasets.

LinearDiscriminantAnalysis: [["SMOTE", SMOTE(random_state=RANDOM_STATE)],

["scaler", RobustScaler()],

["classifier", LinearDiscriminantAnalysis()]]

param_grid = {

"classifier__solver ": ["svd ", "lsqr", "eigen"],

"classifier__shrinkage": (0, 1, 0.01)}

Logistic regression: [["SMOTE", SMOTE(random_state=RANDOM_STATE)],

["preprocessor", RobustScaler()],

["classifier", LogisticRegression(random_state=RANDOM_STATE,

penalty= "l1", solver= "liblinear", fit_intercept=True,

class_weight="balanced")]]

param_grid = {

"classifier__C":[0.001, 0.01, 0.1, 1, 10, 100]}

LinearSVC: [["SMOTE", SMOTE(random_state=RANDOM_STATE)],

["scaler", RobustScaler()],

["classifier", LinearSVC(fit_intercept=True, max_iter = 10000,

class_weight="balanced", random_state=RANDOM_STATE)]]

param_grid = {

"classifier__penalty": ("l1", "l2")}

Bagging Classifier: [["SMOTE", SMOTE(random_state=RANDOM_STATE)],

["scaler", RobustScaler()],

["classifier", BaggingClassifier(random_state=RANDOM_STATE)]]

Extra Tree Classifier: [["SMOTE", SMOTE(random_state=RANDOM_STATE)],

["scaler", RobustScaler()],

["classifier", ExtraTreesClassifier()]]

param_grid = {

"classifier__criterion": ("gini","entropia", "log_loss")}

Evaluating a pipeline: We employed scikit-learn classification reports, presenting performance comparisons for five machine learning classifiers (see S5 Table [30]). These comparisons included standard metrics such as balanced accuracy, ROC AUC, F1-score, and geometric mean (G-mean). Subsequently, resampling was performed by calling the “fit” method; this ensured that the number of samples in “y_pred” matched the number in “y_test” [102].

The Linear Discriminant Analysis (LDA) classifier delivered the best performance among all methods tested. LDA is known for making effective predictions even when assumptions regarding the distribution of each input variable are violated, as observed in our datasets [cf. 108]. Therefore, we chose LDA to implement the Local Interpretable Model-Agnostic Explanation (LIME) and explain the determinants of research publication quality.

Although logistic regression performed well regardless of the data distribution, its performance in our study was lower than that of LDA [cf. 108].

This superior performance of LDA aligns with Hakkoum et al.’s findings [109], who demonstrated that LIME performs well on linear machine learning models.

LDA achieved a higher average percentage of correct classification than logistic regression and the three other methods, as confirmed by higher average values of performance metrics such as ROC AUC, F1-score, G-mean, and Precision_Recall (see S5 Table, [30]).

The LIME algorithm, published by Ribeiro et al. [110], enhances machine learning classifiers by providing insight into the determinants of research publication quality.

It was implemented in Python using the LIME package to generate local explanations for the models. In this study, the constructed models explain tabular data [cf. 110–112].

Model-agnostic explanation systems such as LIME offer a framework for interpretability that allows for flexibility in the selection of models and user expertise [cf. 113]. Consequently, “a system designer who understands why their model is making predictions is certainly better equipped to improve it by means of feature engineering, parameter tuning, or even by replacing the model with a different one” [113, p. 91].

Following Ribeiro et al. [114], who chose a minimum probability of 0.95, we set it slightly upward to 0.96, with a maximum of 1.

Selecting local models with a minimum probability of 0.96 enables us to identify the most probable explanations for the behavior of the original model, ensuring greater accuracy and interpretability.

## Results

**Fig 2:**
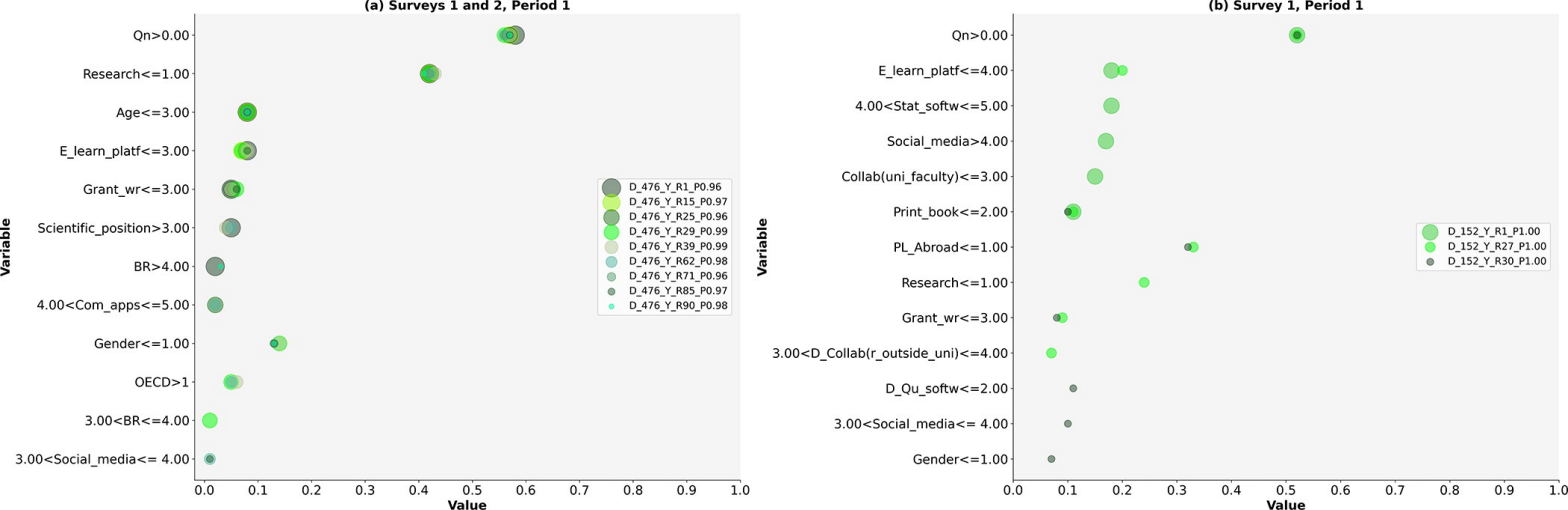
a and b. The Scatterplots of model variables that determine the increase in research publication quality (Period 1).

**Fig 3:**
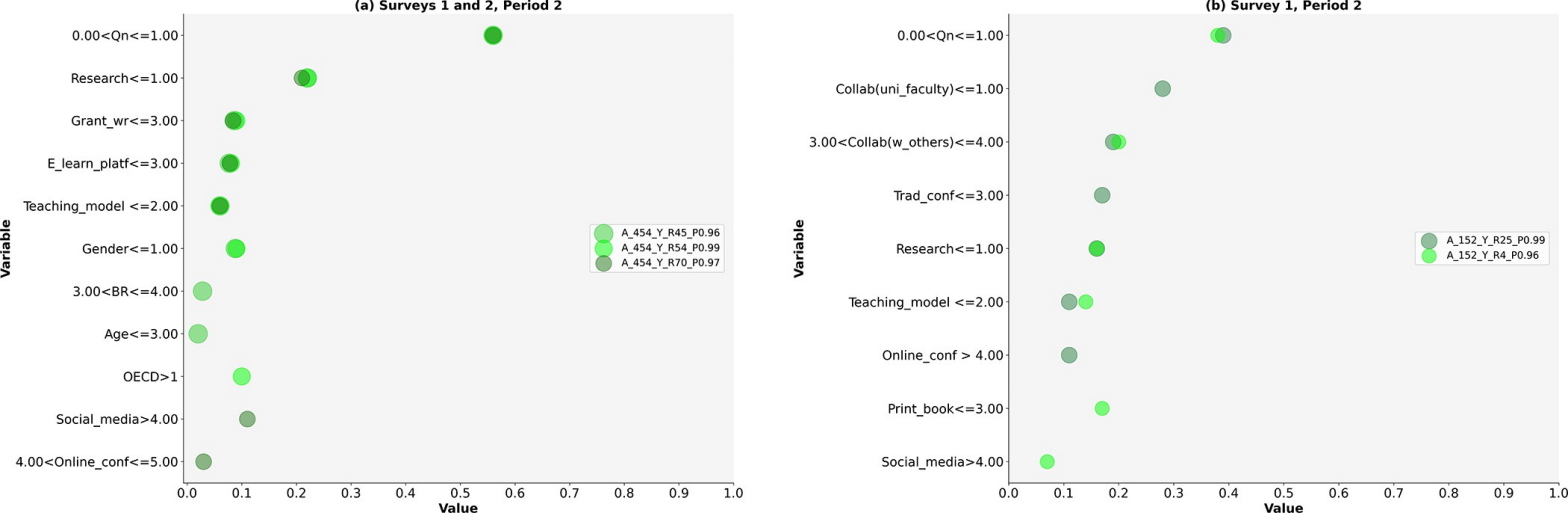
a and b. The Scatterplots of model variables that determine the increase in research publication quality (Period 2).

**Fig 4:**
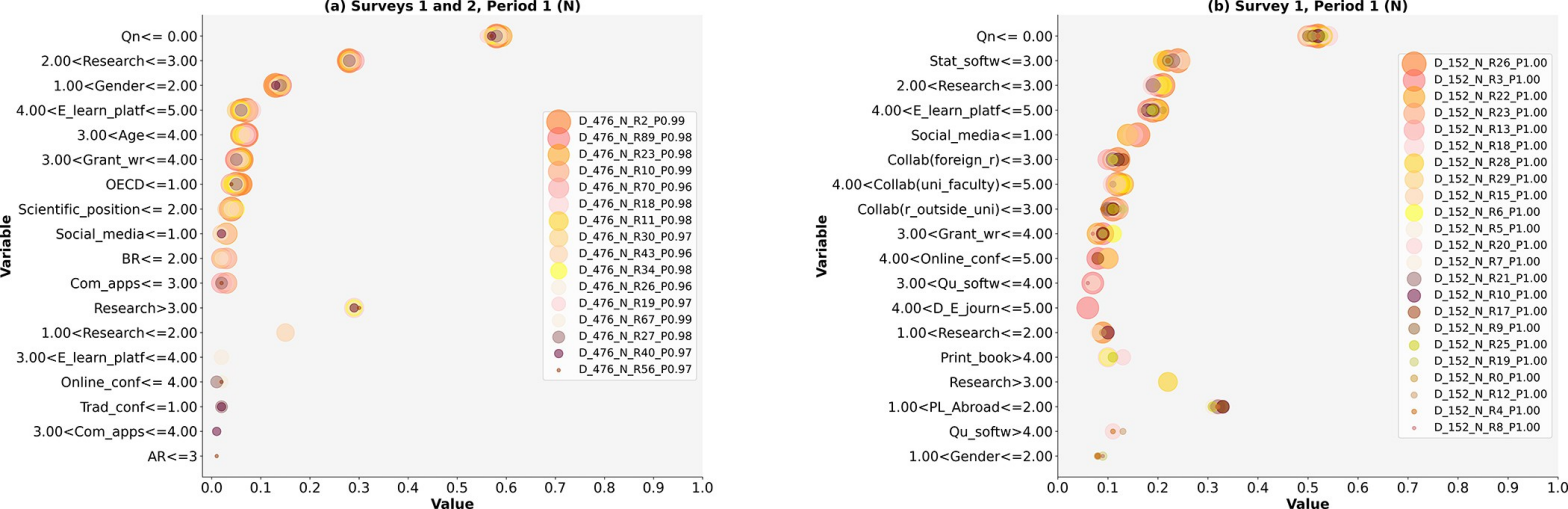
a and b. The Scatterplots of model variables that determine the maintenance of research publication quality (Period 1).

**Fig 5:**
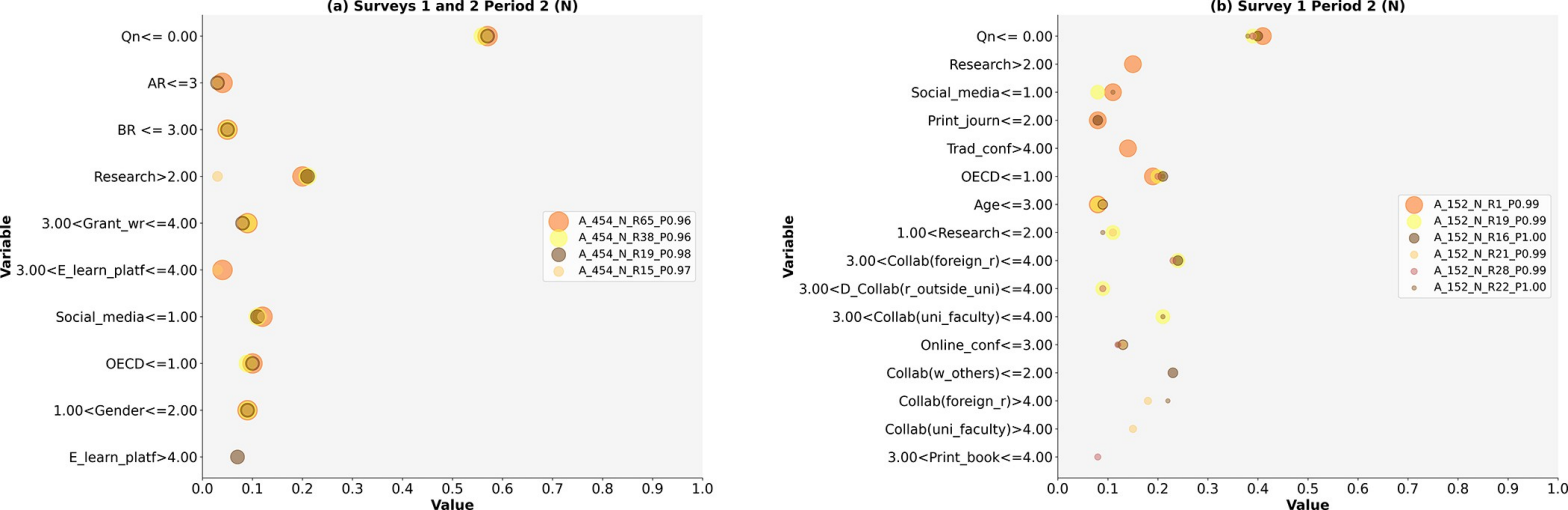
a and b. The Scatterplots of model variables that determine the maintenance of research publication quality (Period 2).

Based on LIME, we answered research questions RQ1 and RQ2 and tested nine hypotheses. The results are presented in Figs 2A–5B and Tables 4–8. This study identifies diverse models of research activities and the use of ICT by academic scientists to increase (Y) or maintain (N) research publication quality in both periods: during the pandemic (Period 1) and after the pandemic (Period 2). The selected models based on Surveys 1 and 2 had a high probability of occurrence (P) ranging from 0.96 to 1.00 (see Figs 2A–5B, [cf. 67]).

**Table 4:** Determinants of models for the increase in the quality of research publications (Period 1).

	Surveys 1 and 2										Survey 1		
Model Variable	D_476_Y_R1_P0.96	D_476_Y_R15_P0.97	D_476_Y_R25_P0.96	D_476_Y_R29_P0.99	D_476_Y_R39_P0.99	D_476_Y_R62_P0.98	D_476_Y_R71_P0.96	D_476_Y_R85_P0.97	D_476_Y_R90_P0.98		D_152_Y_R1_P1.00	D_152_Y_R27_P1.00	D_152_Y_R30_P1.00
Qn>0.00	0.58	0.57	0.57	0.56	0.57	0.56	0.56	0.57	0.57		0.52	0.52	0.52
3.00<BR< = 4.00	0.00	0.00	0.00	0.01	0.00	0.00	0.00	0.00	0.00		0.00	0.00	0.00
BR>4.00	0.02	0.00	0.00	0.00	0.00	0.00	0.00	0.00	0.03		0.00	0.00	0.00
Research< = 1.00	0.42	0.42	0.42	0.42	0.43	0.42	0.42	0.00	0.41		0.00	0.24	0.00
Grant_wr< = 3.00	0.05	0.00	0.05	0.06	0.05	0.00	0.00	0.06	0.00		0.00	0.09	0.08
4.00<Com_apps< = 5.00	0.00	0.00	0.02	0.00	0.00	0.02	0.00	0.00	0.00		0.00	0.00	0.00
E_learn_platf< = 3.00	0.08	0.07	0.00	0.07	0.08	0.00	0.00	0.08	0.00		0.00	0.00	0.00
E_learn_platf< = 4.00	0.00	0.00	0.00	0.00	0.00	0.00	0.00	0.00	0.00		0.18	0.20	0.00
4.00<Stat_softw< = 5.00	0.00	0.00	0.00	0.00	0.00	0.00	0.00	0.00	0.00		0.18	0.00	0.00
Qu_softw< = 2.00	0.00	0.00	0.00	0.00	0.00	0.00	0.00	0.00	0.00		0.00	0.00	0.11
3.00<Social_media< = 4.00	0.00	0.00	0.00	0.00	0.00	0.01	0.00	0.01	0.00		0.00	0.00	0.10
Social_media>4.00	0.00	0.00	0.00	0.00	0.00	0.00	0.00	0.00	0.00		0.17	0.00	0.00
Print_book< = 2.00	0.00	0.00	0.00	0.00	0.00	0.00	0.00	0.00	0.00		0.11	0.11	0.10
3.00<Collab(r_outside_uni)< = 4.00	0.00	0.00	0.00	0.00	0.00	0.00	0.00	0.00	0.00		0.00	0.07	0.00
Collab(uni_faculty)< = 3.00	0.00	0.00	0.00	0.00	0.00	0.00	0.00	0.00	0.00		0.15	0.00	0.00
OECD>1	0.00	0.00	0.00	0.05	0.06	0.05	0.00	0.00	0.00		0.00	0.00	0.00
PL_Abroad< = 1.00	0.00	0.00	0.00	0.00	0.00	0.00	0.00	0.00	0.00		0.00	0.33	0.32
Age< = 3.00	0.08	0.08	0.08	0.08	0.00	0.08	0.08	0.08	0.08		0.00	0.00	0.00
Scientific_position>3.00	0.05	0.00	0.00	0.00	0.04	0.05	0.00	0.00	0.00		0.00	0.00	0.00
Gender< = 1.00	0.00	0.00	0.00	0.14	0.14	0.00	0.13	0.13	0.13		0.00	0.00	0.07

**Table 5:** Determinants of models for the increase in the quality of research publications (Period 2).

	Surveys 1 and 2			Survey 1	
Model Variable	A_454_Y_R45_P0.96	A_454_Y_R54_P0.99	A_454_Y_R70_P0.97	A_152_Y_R25_P0.99	A_152_Y_R4_P0.96
0.00<Qn< = 1.00	0.56	0.56	0.56	0.39	0.38
3.00<BR< = 4.00	0.03	0.00	0.00	0.00	0.00
Research< = 1.00	0.22	0.22	0.21	0.16	0.16
Grant_wr< = 3.00	0.09	0.09	0.08	0.00	0.00
E_learn_platf< = 3.00	0.08	0.08	0.08	0.00	0.00
Social_media>4.00	0.00	0.00	0.11	0.00	0.07
Print_book< = 3.00	0.00	0.00	0.00	0.00	0.17
Online_conf > 4.00	0.00	0.00	0.00	0.11	0.00
4.00<Online_conf< = 5.00	0.00	0.00	0.03	0.00	0.00
Trad_conf< = 3.00	0.00	0.00	0.00	0.17	0.00
Collab(uni_faculty)< = 1.00	0.00	0.00	0.00	0.28	0.00
3.00<Collab(w_others)< = 4.00	0.00	0.00	0.00	0.19	0.20
OECD>1	0.00	0.10	0.00	0.00	0.00
Age< = 3.00	0.02	0.00	0.00	0.00	0.00
Teaching_model < = 2.00	0.06	0.06	0.06	0.11	0.14
Gender< = 1.00	0.09	0.09	0.00	0.00	0.00

**Table 6:** Determinants of models for the maintenance of research publication quality (Surveys 1 and 2 for Period 1).

	Surveys 1 and 2															
Model Variable	D_476_N_R56_P0.97	D_476_N_R40_P0.97	D_476_N_R27_P0.98	D_476_N_R67_P0.99	D_476_N_R19_P0.97	D_476_N_R26_P0.96	D_476_N_R34_P0.98	D_476_N_R43_P0.96	D_476_N_R30_P0.97	D_476_N_R11_P0.98	D_476_N_R18_P0.98	D_476_N_R70_P0.96	D_476_N_R10_P0.99	D_476_N_R23_P0.98	D_476_N_R89_P0.98	D_476_N_R2_P0.99
Qn< = 0.00	0.57	0.57	0.58	0.58	0.56	0.59	0.57	0.58	0.58	0.58	0.58	0.58	0.58	0.59	0.58	0.58
BR< = 2.00	0.00	0.00	0.00	0.02	0.03	0.00	0.00	0.02	0.02	0.00	0.00	0.02	0.03	0.00	0.00	0.00
AR< = 3	0.01	0.00	0.00	0.00	0.00	0.00	0.00	0.00	0.00	0.00	0.00	0.00	0.00	0.00	0.00	0.00
1.00<Research< = 2.00	0.00	0.00	0.00	0.00	0.00	0.00	0.00	0.15	0.00	0.00	0.00	0.00	0.00	0.00	0.00	0.00
2.00<Research< = 3.00	0.00	0.00	0.28	0.00	0.29	0.28	0.00	0.00	0.28	0.28	0.00	0.29	0.28	0.28	0.28	0.28
Research>3.00	0.30	0.29	0.00	0.29	0.00	0.00	0.29	0.00	0.00	0.00	0.29	0.00	0.00	0.00	0.00	0.00
3.00<Grant_wr< = 4.00	0.00	0.00	0.05	0.00	0.05	0.06	0.00	0.05	0.06	0.06	0.00	0.05	0.05	0.06	0.05	0.06
Com_apps< = 3.00	0.02	0.00	0.02	0.00	0.03	0.00	0.00	0.00	0.00	0.00	0.00	0.02	0.03	0.00	0.00	0.00
3.00<Com_apps< = 4.00	0.00	0.01	0.00	0.00	0.00	0.00	0.00	0.00	0.00	0.00	0.00	0.00	0.00	0.00	0.00	0.00
3.00<E_learn_platf< = 4.00	0.00	0.00	0.00	0.00	0.00	0.02	0.00	0.00	0.00	0.00	0.00	0.00	0.00	0.00	0.00	0.00
4.00<E_learn_platf< = 5.00	0.00	0.00	0.06	0.06	0.06	0.00	0.06	0.07	0.05	0.06	0.08	0.00	0.06	0.06	0.06	0.07
Social_media< = 1.00	0.00	0.02	0.00	0.02	0.00	0.00	0.00	0.02	0.00	0.00	0.03	0.00	0.00	0.03	0.00	0.00
Online_conf< = 4.00	0.02	0.00	0.01	0.02	0.00	0.00	0.00	0.00	0.00	0.00	0.00	0.00	0.00	0.00	0.00	0.00
Trad_conf< = 1.00	0.00	0.02	0.02	0.00	0.00	0.00	0.00	0.00	0.00	0.00	0.00	0.00	0.00	0.00	0.00	0.00
OECD< = 1.00	0.04	0.00	0.05	0.05	0.00	0.05	0.04	0.00	0.05	0.05	0.04	0.05	0.04	0.06	0.06	0.05
3.00<Age< = 4.00	0.00	0.00	0.00	0.00	0.07	0.07	0.06	0.06	0.00	0.00	0.07	0.07	0.06	0.06	0.07	0.07
Scientific_position< = 2.00	0.00	0.00	0.00	0.00	0.05	0.04	0.05	0.03	0.04	0.04	0.00	0.00	0.00	0.00	0.00	0.04
1.00<Gender< = 2.00	0.00	0.13	0.14	0.13	0.00	0.00	0.00	0.14	0.14	0.14	0.00	0.00	0.13	0.13	0.14	0.13

**Table 7:** Determinants of models for the maintenance of research publication quality (Survey 1 for Period 1).

	Survey 1																						
Model Variable	D_152_N_R26_P1.00	D_152_N_R3_P1.00	D_152_N_R22_P1.00	D_152_N_R23_P1.00	D_152_N_R13_P1.00	D_152_N_R18_P1.00	D_152_N_R28_P1.00	D_152_N_R29_P1.00	D_152_N_R15_P1.00	D_152_N_R6_P1.00	D_152_N_R5_P1.00	D_152_N_R20_P1.00	D_152_N_R7_P1.00	D_152_N_R21_P1.00	D_152_N_R10_P1.00	D_152_N_R17_P1.00	D_152_N_R9_P1.00	D_152_N_R25_P1.00	D_152_N_R19_P1.00	D_152_N_R0_P0.99	D_152_N_R12_P1.00	D_152_N_R4_P1.00	D_152_N_R8_P1.00
Qn< = 0.00	0.52	0.51	0.52	0.50	0.51	0.54	0.53	0.51	0.50	0.51	0.50	0.53	0.53	0.52	0.52	0.51	0.50	0.53	0.51	0.50	0.52	0.52	0.52
1.00<Research< = 2.00	0.00	0.00	0.09	0.09	0.00	0.00	0.00	0.00	0.00	0.00	0.00	0.00	0.08	0.00	0.10	0.00	0.00	0.00	0.00	0.09	0.00	0.00	0.09
2.00<Research< = 3.00	0.21	0.20	0.00	0.00	0.00	0.19	0.00	0.21	0.20	0.21	0.00	0.19	0.00	0.19	0.00	0.00	0.00	0.00	0.00	0.00	0.20	0.00	0.00
Research>3.00	0.00	0.00	0.00	0.00	0.00	0.00	0.22	0.00	0.00	0.00	0.00	0.00	0.00	0.00	0.00	0.00	0.00	0.00	0.00	0.00	0.00	0.00	0.00
3.00<Grant_wr< = 4.00	0.00	0.09	0.08	0.00	0.00	0.00	0.00	0.00	0.00	0.11	0.00	0.00	0.08	0.00	0.09	0.09	0.00	0.00	0.09	0.09	0.00	0.00	0.07
4.00<E_learn_platf< = 5.00	0.19	0.20	0.20	0.18	0.18	0.20	0.20	0.19	0.19	0.00	0.00	0.19	0.18	0.19	0.18	0.19	0.19	0.19	0.19	0.21	0.17	0.00	0.00
Stat_softw< = 3.00	0.24	0.00	0.22	0.22	0.23	0.00	0.21	0.00	0.23	0.00	0.25	0.00	0.00	0.23	0.00	0.00	0.22	0.00	0.22	0.00	0.00	0.22	0.00
3.00<Qu_softw< = 4.00	0.00	0.07	0.00	0.00	0.00	0.00	0.00	0.00	0.00	0.00	0.00	0.00	0.07	0.00	0.00	0.00	0.00	0.00	0.00	0.00	0.00	0.00	0.06
Qu_softw>4.00	0.00	0.00	0.00	0.00	0.00	0.00	0.00	0.00	0.00	0.00	0.00	0.11	0.00	0.00	0.00	0.00	0.00	0.00	0.00	0.00	0.13	0.11	0.00
Social_media< = 1.00	0.16	0.00	0.14	0.00	0.16	0.15	0.00	0.14	0.00	0.00	0.00	0.00	0.00	0.00	0.00	0.00	0.00	0.00	0.00	0.00	0.00	0.00	0.00
4.00<E_journ< = 5.00	0.00	0.06	0.00	0.00	0.00	0.00	0.00	0.00	0.00	0.00	0.00	0.00	0.00	0.00	0.00	0.00	0.00	0.00	0.00	0.00	0.00	0.00	0.00
Print_book>4.00	0.00	0.00	0.00	0.00	0.00	0.10	0.00	0.00	0.00	0.10	0.00	0.13	0.10	0.00	0.00	0.00	0.00	0.11	0.00	0.00	0.00	0.00	0.00
4.00<Online_conf< = 5.00	0.00	0.08	0.00	0.10	0.09	0.10	0.10	0.10	0.10	0.00	0.00	0.00	0.00	0.00	0.00	0.08	0.00	0.00	0.00	0.00	0.00	0.00	0.00
Collab(foreign_r)< = 3.00	0.12	0.00	0.00	0.11	0.10	0.00	0.00	0.00	0.00	0.00	0.11	0.00	0.00	0.11	0.12	0.13	0.12	0.11	0.11	0.11	0.00	0.00	0.00
Collab(r_outside_uni)< = 3.00	0.11	0.00	0.00	0.12	0.11	0.00	0.00	0.00	0.12	0.00	0.11	0.00	0.00	0.10	0.11	0.11	0.10	0.11	0.13	0.12	0.00	0.09	0.00
4.00<Collab(uni_faculty)< = 5.00	0.12	0.00	0.13	0.00	0.00	0.11	0.12	0.12	0.00	0.13	0.00	0.12	0.00	0.00	0.00	0.00	0.00	0.00	0.00	0.00	0.11	0.00	0.00
1.00<PL_Abroad< = 2.00	0.00	0.00	0.00	0.00	0.00	0.00	0.00	0.00	0.00	0.00	0.00	0.32	0.31	0.32	0.33	0.33	0.33	0.31	0.00	0.00	0.31	0.32	0.32
1.00<Gender< = 2.00	0.00	0.00	0.00	0.00	0.00	0.00	0.00	0.00	0.00	0.00	0.00	0.00	0.00	0.00	0.00	0.00	0.00	0.00	0.09	0.08	0.08	0.08	0.09

**Table 8:** Determinants of models for the maintenance of research publication quality (Period 2).

	Surveys 1 and 2					Survey 1					
Model Variable	A_454_N_R15_P0.97	A_454_N_R19_P0.98	A_454_N_R38_P0.96	A_454_N_R65_P0.96		A_152_N_R22_P1.00	A_152_N_R28_P0.99	A_152_N_R21_P0.99	A_152_N_R16_P1.00	A_152_N_R19_P0.99	A_152_N_R1_P0.99
Qn< = 0.00	0.57	0.57	0.56	0.57		0.38	0.39	0.39	0.40	0.39	0.41
AR< = 3	0.03	0.03	0.00	0.04		0.00	0.00	0.00	0.00	0.00	0.00
BR < = 3.00	0.05	0.05	0.05	0.05		0.00	0.00	0.00	0.00	0.00	0.00
1.00<Research< = 2.00	0.00	0.00	0.00	0.00		0.09	0.00	0.11	0.00	0.11	0.00
Research>2.00	0.03	0.21	0.21	0.20		0.00	0.00	0.00	0.00	0.00	0.15
3.00<Grant_wr< = 4.00	0.08	0.08	0.09	0.09		0.00	0.00	0.00	0.00	0.00	0.00
3.00<E_learn_platf< = 4.00	0.03	0.00	0.00	0.04		0.00	0.00	0.00	0.00	0.00	0.00
E_learn_platf>4.00	0.00	0.07	0.00	0.00		0.00	0.00	0.00	0.00	0.00	0.00
Social_media< = 1.00	0.12	0.11	0.11	0.12		0.11	0.00	0.00	0.00	0.08	0.11
Print_journ< = 2.00	0.00	0.00	0.00	0.00		0.00	0.00	0.00	0.08	0.00	0.08
3.00<Print_book< = 4.00	0.00	0.00	0.00	0.00		0.00	0.08	0.00	0.00	0.00	0.00
Online_conf< = 3.00	0.00	0.00	0.00	0.00		0.12	0.12	0.13	0.13	0.00	0.00
Trad_conf>4.00	0.00	0.00	0.00	0.00		0.00	0.00	0.00	0.00	0.00	0.14
3.00<Collab(foreign_r)< = 4.00	0.00	0.00	0.00	0.00		0.00	0.23	0.00	0.24	0.24	0.00
Collab(foreign_r)>4.00	0.00	0.00	0.00	0.00		0.22	0.00	0.18	0.00	0.00	0.00
3.00<Collab(r_outside_uni)< = 4.00	0.00	0.00	0.00	0.00		0.00	0.09	0.00	0.00	0.09	0.00
3.00<Collab(uni_faculty)< = 4.00	0.00	0.00	0.00	0.00		0.21	0.00	0.00	0.00	0.21	0.00
Collab(uni_faculty)>4.00	0.00	0.00	0.00	0.00		0.00	0.00	0.15	0.00	0.00	0.00
Collab(w_others)< = 2.00	0.00	0.00	0.00	0.00		0.00	0.00	0.00	0.23	0.00	0.00
OECD< = 1.00	0.10	0.10	0.09	0.10		0.21	0.20	0.00	0.21	0.20	0.19
Age< = 3.00	0.00	0.00	0.00	0.00		0.00	0.00	0.09	0.09	0.08	0.08
1.00<Gender< = 2.00	0.09	0.09	0.09	0.09		0.00	0.00	0.00	0.00	0.00	0.00

We analyzed the determinants of the models that increase probability.

We questioned the possibility of providing a negative answer to RQ1. The evidence from our study undeniably demonstrates that the activity models differ between periods among academic scientists who increase or maintain research publication quality. The detailed results are presented below.

### Models for increasing quality (Y)

A detailed answer to the research question RQ1 requires testing hypotheses H1a–H1f. The identified models provide evidence of significant differences between the research activities of academic scientists who increased quality and those who, at most, maintained it. The differences are as follows:

Period 1

Diverse activities, including ICT use, contribute to increasing the quality of research publications (see Table 4). We identified nine models based on Surveys 1 and 2 (D_476_Y, Table 4) and three additional models based solely on Survey 1 (D_152_Y, Table 4). Four models (Y_R15_P0.97, Y_R25_P0.96, Y_R1_P0.96, and Y_R62_P0.98) describe the activities of academic scientists aged 49 or younger, including one (Y_R1_P0.96) for all scientific positions except graduate students, lecturers, and assistant professors. Four models (Y_R29_P0.99, Y_R71_P0.96, Y_R85_P0.97, and Y_R90_P0.98) were constructed for female academic scientists aged 49 or younger, with one model specifically for female activities in all science disciplines except the social sciences (Y_R29_P0.99). The model based on Surveys 1 and 2 (Y_R39_P0.99) describes the activities of women aged 49 or younger who are graduate students, lecturers, or assistant professors in all scientific disciplines except the social sciences, which contribute to an increase in quality. A model based on the results of Survey 1 (Y_R1_P1.00) explains research activities irrespective of gender, age, scientific position, science discipline, or teaching model (see Fig 2B). However, the second (Y_R27_P1.00) and third (Y_R30_P1.00) models emphasize the roles of Polish academic scientists and/or female scientists in increasing research publication quality. Each model included at least two research activities, with a maximum of four activities presented in both models.

Our study demonstrates the significance of RQ1, as the models reveal a diversity of activities (Table 4). In some cases, being female and under 49 years of age was important for increasing research publication quality, while in others, holding any scientific position other than that of graduate student, lecturer, or assistant professor was important. In one model, gender, academic position, science discipline, country, and age were found to be irrelevant.

Period 2

The variety of activity models aimed at increasing research publication quality is reduced in Period 2 compared to Period 1 (cf. Tables 4 and 5, Figs 2A–3B). Five models with their determinants were identified: three based on the results from Surveys 1 and 2 (A_454_Y) and two based on Survey 1 alone (A_152_Y), where all teaching models except online teaching are crucial. One model (Y_R70_P0.97) reveals research activities irrespective of gender, age, scientific position, or science discipline (see Table 5). The second model (A_454_Y_R45_P0.96) describes the activities of female academics up to 49 years old. The third model (A_454_Y_R54_P0.99) identifies the research activities of female academics across all science disciplines, excluding the social sciences. Additionally, two models based on the results of Survey 1 (152_Y_R25_P0.99 and 152_Y_R4_P0.96) describe academic scientists’ research activities irrespective of gender, age, scientific position, or science discipline. Each model comprises at least three research activities (see Fig 3B).

### Research activities on the increase in quality (Y)

The identified models indicate a reduced variety of activities among academic scientists who increased their research publication quality in Period 2 compared to Period 1 (Figs 2A–3B).

Period 1

Academic scientists who increased the quality of research publications were a minority. They prioritized the number of research publications (see Fig 2A and 2B, Table 4) and spent most of their time on research, with the exception of three models. Furthermore, some academic scientists placed grant writing third in importance. Collaboration with faculty scientists was crucial in only one model (see Fig 2B).

Period 2

Academic scientists aiming to increase the quality of their research publications in Period 2 also represented a minority. The number of research publications remains the highest priority (a maximum weight = 0.56, Fig 3A and 3B, Table 5). Collaboration with others is crucial for increasing quality. These results partially support hypothesis H1f.

This study partially supports the first hypothesis H1a, as the increase in research publication quantity pertains solely to the enhancement of research publication quality in both periods. Furthermore, gender or science discipline influences on activity models.

The importance of collaboration with scientists at faculties was higher than that of conducting research; however, research remains more significant than grant writing. Grant writing ranks low in both periods.

The evidence partially supports hypothesis H1b, as research was prioritized only in Period 1, particularly by female academic scientists in some activity models. Furthermore, research refers solely to increasing publication quality. This study rejects the third hypothesis (H1c) due to the low ranking of grant writing among academic scientists who increase or, at most, maintain the quality of research publications. Additionally, some academic scientists do not engage in collaboration, while others participate in at least one form of collaboration. The results partially support hypothesis H1e, as collaboration with domestic scientists was crucial for increasing quality in Period 1. Detailed results are presented in Table 4 and Fig 2B.

### Models for maintaining quality (N)

The evidence from this study confirms the greater variety of activities among academic scientists who maintained quality in Period 1 (i.e., 16 models based on Surveys 1 and 2, and 23 models based on Surveys 1) than in Period 2 (Tables 6–8). We selected models in which each of the 39 models had a high probability of occurrence (P> = 0.96 or a maximum of 1.00).

Period 1

Based on the results of Surveys 1 and 2, one model identified the research activities of academic scientists in the social sciences, irrespective of gender, age, or scientific position (D_476_N_R56_P0.97; Table 6). In the second model, the activities of male academic scientists (D_476_N_R40_P0.97) were distinguished. However, the activities of this group of academic scientists differed from those of other male academics in the social sciences (i.e., D_476_R27_P0.98, D_476_R67_P0.99). Furthermore, one model describes activities by Ph.D. students, lecturers, and assistant professors aged 50–68, irrespective of science discipline and gender (D_476_N_R19_P0.97).

The following two models concern the activities of the same group of academic scientists but in social sciences (D_476_N_R26_P0.96, D_476_N_R34_P0.98). Male Ph.D. students or lecturers aged 50–68 years performed more activities that contributed to maintaining the quality of publications (D_476_N_R43_P0.96). Their counterparts in social sciences differed in their activities (D_476_N_R30_P0.97, D_476_N_R11_P0.98). Each model includes at least two activities, and five models contain a maximum of five activities (see Table 6).

Other models demonstrate varied research activities of academic scientists aged 50–68 in the social sciences (D_476_N_R18_P0.98, D_476_N_R70_P0.96). Three additional models explain the activities of the male group of scientists in the social sciences. Finally, the last model, based on Surveys 1 and 2, applies to scientific positions, such as Ph.D. students, lecturers, and assistant professors in the social sciences (D_476_N_R2_P0.99, Table 6). Based on Survey 1, 11 activity models were identified, irrespective of age, scientific position, and science discipline (i.e., D_152_N_R26_P1.00, D_152_N_R3_P1.00, D_152_N_R22_P1.00, D_152_N_R23_P1.00, D_152_N_R13_P1.00, D_152_N_R18_P1.00, D_152_N_R28_P1.00, D_152_N_R29_P1.00, D_152_N_R15_P1.00, D_152_N_R6_P1.00, D_152_N_R5_P1.00, Table 7). These models contain a minimum of three activities and a maximum of five. Seven models describe activities by academic scientists excluding Poland (D_152_N_R20_P1.00, D_152_N_R7_P1.00, D_152_N_R21_P1.00, D_152_N_R10_P1.00, D_152_N_R17_P1.00, D_152_N_R9_P1.00, D_152_N_R25_P1.00). Two models describe activities by male academic scientists (D_152_N_R19_P1.00, D_152_N_R0_P0.99), and the last three models concern males, excluding Poland (D_152_N_R12_P1.00, D_152_N_R4_P1.00, and D_152_N_R8_P1.00).

We address RQ1 because the described models identify the most diverse activities. In most models of maintaining quality, gender, academic position, science discipline, region, and age are considered unimportant. In some cases, being a male academic scientist and/or being aged 50–68 in the social sciences has significant implications for maintaining quality. Other models describe activities for all scientific positions except for doctoral students and faculty, or only for these groups.

Period 2

This study provides evidence for a smaller variety of activity models—four models based on Surveys 1 and 2, and six models based on Survey 1 (Table 8)–compared to Period 1. Four models explain the activities of male academics in the social sciences (A_454_N_R15_P0.97, A_454_N_R19_P0.98, A_454_N_R38_P0.96, and A_454_N_R65_P0.96). However, the second group of models includes more activities than the models based on Surveys 1 and 2. One model pertains to academic scientists, irrespective of discipline and gender (A_152_N_R21_P0.99). In the last three models, age (up to 39 years) and the social science discipline have a significant influence on the maintenance of research publication quality (Table 8). The last model contains the fewest research activities, referring to social studies and age up to 49 years (A_152_N_R1_P0.99).

### Research activities for maintaining quality (N)

The evidence of our study conclusively demonstrates that activities vary the most among models for academic scientists who maintain quality in both periods. Male gender influences differences in research activity related to maintaining research publication quality.

Period 1

In most models, research was not a priority activity, and grant writing consistently ranked last (11 models based on Surveys 1 and 2; Table 6). One model focuses on maintaining both basic and applied research.

In the 11 models based on Survey 1, collaboration with foreign scientists and domestic scientists outside their university was neither strongly emphasized nor disregarded (Table 7). Collaboration with university or faculty scientists was the least important for academic scientists (8 of 23 models). However, this type of collaboration was crucial for male academic scientists, except those from Poland.

Period 2

In all models, quantity is crucial (Table 8). Research activities are ranked third or lower. Writing grants ranked last in the activity ranking (four models based on Surveys 1 and 2, Fig 5A). Research occasionally focuses on basic and/or applied research.

Collaboration with university or faculty academic scientists and domestic scientists outside their university is crucial for academic scientists in the social sciences or those up to 49 years old in the social sciences, according to Survey 1-based models (Fig 5B). Only one model includes no collaboration (A_152_N_R1_P0.99). In most models, academic scientists collaborate with two groups of scientists.

The results partially support hypothesis H1d, as collaboration with foreign scientists is crucial in some models for maintaining research publication quality, but only in Period 2. However, collaboration with domestic scientists is vital for maintaining quality in both periods (see Survey 1); therefore, the evidence partially supports hypothesis H1e as well.

### The use of ICT in research

A detailed answer to the research question RQ2 requires testing hypotheses H2a, H2b, and H2c. We partially questioned their ability to provide a positive response to RQ2. Our evidence demonstrates conclusively that only some academic scientists use multiple (minimum of two) ICTs often or very often to increase quality. Their diversity was small in Period 1 (D_152_Y_R1_P1.00, D_152_Y_R27_P1.00) and non-existent in Period 2 (one model A_454_Y_R70_P0.97). In contrast, some academic scientists used many ICTs (i.e., 11 models among the 39 models for Period 1) often or very often to maintain quality. We did not find a model containing many ICTs for this group of academic scientists during Period 2. Gender (male) also has a significant effect on these models. However, academic scientists differ significantly in their frequency of ICT use (most identified models).

The results of this study suggest that H2a is partially supported because only some models include new communication technology, irrespective of gender and science discipline. To increase quality in both periods, some academic scientists used social media frequently or very frequently (in 4 of the 12 models for Period 1 and in 2 of the 5 models for Period 2; see Tables 4 and 5). However, half of these models for Period 1 emphasize the importance of all academic disciplines except the social sciences, and one model involves male academic scientists. To maintain quality in both periods, academic scientists choose e-learning platforms (32 models among 39 for Period 1; 4 models among 11 for Period 2); however, most of the models for Period 2 involve the social sciences.

The evidence from this research suggests the rejection of H2b and H2c because the same new or old information technology is not used in both periods often or very often to increase or maintain quality. Detailed results are provided in Tables 4–8.

### The increase in quality using ICT

The identified models indicated the least variety of ICT tools, particularly compared to the models for maintaining quality (cf. Tables 4–8, Figs 2A–5B).

Period 1

Academic scientists often, very often, or at least sometimes selected one technology from ten analyzed new ICTs and three non-digital information technologies to increase the quality of research publications. In the four Surveys 1 and 2-based models, only one ICT tool, namely e-learning platforms used sometimes or less frequently, was selected by academic scientists, and two models included communication apps (Table 4). Academic scientists rarely used questionnaire software (one model, D_152_Y_R30_P1.00, Fig 2B), whereas four models included social media used often or very often (including by women, Fig 2A and 2B), and print books were seldom used (Fig 2B). Some academic scientists used statistical analysis software more often than e-learning platforms or social media (one model, see Fig 2B). They were least likely to use print books in their studies. Academic scientists aged up to 49 chose either a communication app (very often; D_476_Y_R25_P0.96) or an e-learning platform (D_476_Y_R15_P0.97, Fig 2A). Associate professors, retired professors, researchers, and assistants sometimes or less frequently used e-learning platforms in their research. However, for some academic scientists in all scientific disciplines, except for the social sciences, the use of communication apps (very often) and social media (most often) was crucial (D_476_Y_R62_P0.98). In two models, both old and new ICTs were crucial for increasing quality (D_476_Y_R71_P0.96, D_476_Y_R90_P0.98).

Period 2

During this period, a low diversity of ICTs for increasing the quality of research publications was identified (Fig 3A and 3B). Participating in online conferences is rather important for some academic scientists, irrespective of gender, age, or science discipline (454_Y_R70_P0.97; 152_Y_R25_P0.99; see Table 5). Participation in traditional conferences is also important (152_Y_R25_P0.99; Fig 3B). Some academic scientists very often use social media in research (454_Y_R70_P0.97; 152_Y_R4_P0.96; see Fig 3A and 3B) and occasionally choose a print book in research to increase quality (152_Y_R4_P0.96).

### Maintenance of quality using ICT

The activity models showed the most diverse use of ICT tools compared to the models for increasing quality. In Period 1 (Table 6), academic scientists often or very often used a maximum of four ICT tools but in only two models.

Period 1

According to the results of Surveys 1 and 2 (D_N_476), almost all models included at least one type of ICT tool, except for D_476_N_R5_P1.00. Five models included communication apps that were seldom or rarely used (Fig 4A). The use of e-learning platforms (often or very often) was crucial for research purposes. Moreover, academic scientists in the social sciences, mainly male, often participated in online conferences (see Table 6).

Academic scientists often or very often used a maximum of three ICT tools for research (Table 7). The use of social media to maintain quality was not crucial in five models. Furthermore, male academic scientists and those in the social sciences did not participate in traditional conferences. Of the models based on Survey 1 (i.e., D_N_152), nine included e-learning platforms frequently used in research by academic scientists, irrespective of gender, scientific position, or age (Table 7). Eight models included statistical analysis software used sometimes or less often, and male academic scientists often or very often used questionnaire software (two models: 152_N_R12_P1.00, and 152_N_R4_P1.00). Some academic scientists, irrespective of gender, position, or country, chose online conferences (i.e., very often in seven models, Table 7 and Fig 4B). In only two models (152_N_R18_P1.00, 152_N_R6_P1.00), academic scientists, irrespective of gender, country, age, or scientific position, frequently used print books.

Period 2

Irrespective of social position, some male academic scientists in the social sciences often or more frequently used e-learning platforms (three models: 454_N_R15_P0.97, 454_N_R19_P0.98, and 454_N_R65_P0.96, Table 8). Participating in online conferences is neither important nor unimportant for maintaining quality for some academic scientists in the social sciences, irrespective of gender (152_N_R22_P1.00; 152_N_R28_P0.99; 152_N_R16_P1.00). Some academic scientists do not use social media in their research (three models based on Survey 1 and four models based on Surveys 1 and 2). Conversely, some often use print books (152_N_R28_P0.99). For academic scientists up to 49 years old, online conferences are neither important nor unimportant for maintaining quality (152_N_R16_P1.00, 152_N_R21_P0.99). In the social sciences, academic scientists often participate in traditional conferences (152_N_R1_P0.99; see Table 8 and Fig 5B). In two models, academic scientists seldom use print journals in their research (152_N_R16_P1.00, 152_N_R1_P0.99).

## Discussion

Shen et al. [21] have emphasized that insufficient attention has been paid to examining the diversity of behaviors within organizations, particularly in research settings. To address this gap, our study mapped the diversity of research activities and the use of ICTs during and after the pandemic, classifying these activities and tools as determining either the increase or maintenance of research publication quality.

### The relationship between the quantity and quality of research publications

Scientists have long debated the relationship between the quantity and quality of scientific publications. Previous studies suggested that the number of high-quality publications increases proportionally to the total number of publications by scientists. Moreover, the relationship between quantity and quality appeared weaker for lower-quality publications and stronger for higher-quality publications [115].

We explored the quality-quantity relationship in a novel manner, analyzing it as one of several potential determinants aimed at increasing or maintaining the quality of research publications. Our findings indicate that academic scientists increase the number of research publications to enhance quality. However, academic scientists who maintain research publication quality do not change or decrease their number of research publications. Notably, most of the respondents in our study maintained the quality of their research publications. These findings contrast with Rowley and Sbaffi’s [54] assertion that academic scientists publish as many high-quality articles as possible. Instead, our evidence aligns more closely with Michalska-Smith and Allesina’s [116] evidence of the positive relationship between quantity and quality in highly cited publications. However, they observed that a positive relationship exists between publication quality and quantity for the most-cited publications, where higher quality (measured by a higher citation count) is associated with greater publication quantity during years of higher productivity.

Additionally, some of our models align closely with Aguinis et al.’s statement [13] that when facing increased pressures, scientists probably do not increase their knowledge or contributions to their field; rather, their sole benefit is gaining experience in the "publication game."

In conclusion, our study contributes to the ongoing discourse on the quality-quantity relationship in scientific publications. Our findings indicate that research publication quantity is not the sole determinant of research publication quality.

### Role of gender in research publication quality

Our study found that gender differences among academic scientists did not increase the quality of research publications. Therefore, our findings do not fully align with Rowley and Sbaffi’s general statement [54] that women prioritize high-quality publishing. Nonetheless, in some research activity models, individuals identified as female showed increased publication quality. This might stem from the fact that (basic) research conducted by academic scientists identifying as female tends to be associated with a lower average of overall questionable research practices, as observed by Gopalakrishna et al. [3]. Consequently, this suggests that, irrespective of gender, obtaining data and conducting data analysis might require more rigorous attention during peer review.

### Relationship between research collaborations and research publication quality

This study found that collaboration with domestic and/or international academic scientists played an important role in maintaining the quality of research publications, but only among some scientists during both periods. Notably, during the pandemic, collaboration with domestic academic scientists from outside the university was similarly important for increasing research publication quality.

These findings offer a new perspective on academic collaboration, which is often perceived as superior for achieving high research quality [61,62,117].

Furthermore, we found that collaboration with non-academics was crucial for some academic scientists in increasing publication quality after the pandemic. Thus, these results contrast with Lauvås and Steinmo’s [118] assertion that industry-university collaboration does not contribute to high-quality academic publications.

### Mixed role of ICTs in research publication quality

A thorough literature review provides ambiguous evidence regarding the relationship between ICT tools and the quality of research publications. Our findings address this research gap. Our results contrast with those of Alammary et al. [78], who reported that academic scientists’ exposure to e-learning tools during the pandemic positively influenced their perception of e-learning after the pandemic.

Although our evidence supports the conclusions of Ziemba and Eisenbardt [19] and Wartini-Twardowska et al. [18] that e-learning platforms are utilized less after the pandemic and that the use of social media remains at the same level during and after the pandemic, social media is not crucial for research publication quality among academic scientists. Furthermore, the frequent use of social media characterized academic scientists who increased quality in both periods. Conversely, a lack of social media use was typical for academic scientists who maintain research publication quality.

Another ICT tool, statistical analysis software, was used by academic scientists to increase their research publication quality, but only within one research activity model. For some academic scientists who maintained quality, frequent use of questionnaire software during the pandemic in their research was important, as was occasional use of statistical analysis software or use of communication applications (at most, often). This partly supports the finding by Stein and Sim [74] that academic scientists consider ICTs essential in their daily research. However, it is clear from our findings that such generalizations do not apply to all academic scientists. Nevertheless, we recognize the importance responsibly and effectively incorporating these technologies into research processes, as suggested by the authors.

Additionally, we found that individuals who continued to use ICTs (specifically information systems) did not retain their intention to use them to the same extent, contrary to the findings of Naranjo-Zolotov et al. [119].

Our study also provides evidence that some academic scientists in both groups either do not use any ICT tools or use only one type of ICT tool, and they do so only occasionally to increase or maintain the quality of their research publications.

Consequently, our analysis revealed a diverse array of research activity models, distinguishing between the use of ICTs to maintain or increase the quality of research publications. These models demonstrate that the use of ICTs should not be assessed holistically in terms of their contribution to research publication quality. Instead, they must be categorized to establish their specific impact on research publication quality, particularly in terms of increase versus maintenance. Therefore, the findings of Ding et al. [72] regarding the significant increase in productivity using ICTs require further analysis in terms of research activity models to increase or maintain research publication quality.

### Relationship between preferences for the (e-)publication and the quality of research publications

In contrast to Hemminger et al. [24], our study did not observe an increased reliance on e-publications. Instead, the frequent use of either electronic-only resources (including e-journals) or print-only resources (including print books) was crucial for some academic scientists who maintained quality during the pandemic.

The information overload theory offers an explanation for the research activities of academic scientists. A constant stream of information provided in digital formats may lead to a feeling of supersaturation [120]. In the 21st century, characterized by the shift to hyperhistory and the infosphere, individuals and societies rely on information more than ever before. As a result, information overload warrants greater consideration. This was experienced by some academic scientists in both the quality-increasing and quality-maintaining groups, who opted to use print books in their research, particularly after the pandemic.

### Implications for academic scientists and university authorities

As universities strive to enhance the efficiency of their research systems, it is essential to measure publication quality on a scale that reflects the ideal types of activity models. Our approach, which applies Local Interpretable Model-agnostic Explanation (LIME) to evaluate academic scientists’ activities, provides insights into which models most significantly advance a university’s objectives. This approach can guide efforts to advance academic scientists and enhance the overall quality of research publications associated with university departments by identifying underperforming activity models and addressing their limitations.

Our approach facilitates the comprehensive screening of academic scientists’ performance. It is crucial to transparently identify neglected research activities and assess the state of academic scientist’s technological knowledge. Such evaluations can contribute significantly to maintaining high research standards and fulfilling the mission of a university. Additionally, recognizing the diversity among academic scientists underscores that they are not a homogeneous group, which is crucial for managing research publication quality. Leveraging this diversity can enhance the synergies of efforts aimed at improving research publication quality. Universities can benefit from using activity models to analyze research staff behavior and identify the most effective approaches for enhancing publication quality. These models not only boost individual and team synergies but also aid in managing academic scientists by identifying potential weaknesses or policy violations, thereby ensuring a more effective research environment.

As Gorodnichenko et al. [55] pointed out, the outcomes of a modified evaluation require a five-year gap between the incentive for academic scientists to change their activities and the realization of those changes. Therefore, a new survey is essential to provide updated insights into ideal research activity models for quality-maintaining or quality-increasing academic scientists and their impact on achieving university objectives.

## Conclusions

We classified the models of quality-increasing and quality-maintaining research activities, as well as the use of technology in research by academic scientists, to explain how the pandemic affected their behaviors in our study groups. We analyzed sixty-six models and reached the following conclusions:

First, the pandemic led to significant diversity in the behaviors of both groups of academic scientists. Academic scientists who maintained quality exhibited the greatest diversity in research activities and ICT use. By contrast, those who sought to increase research quality were less affected by the pandemic, as their models were less diverse. Identifying activity models was critical, as it provided insight into which research activities and ICTs were prioritized or neglected. We recommend this approach for monitoring the quality of research publications.

Second, academic scientists increased the number of their publications to enhance the quality of their research publications. Maintaining a consistent publication count was necessary to sustain this quality. Irrespective of whether academic scientists prioritize increasing the number of publications or maintaining quality, the number of scientific publications plays a central role in all models of research activities.

Third, women appeared to prioritize increasing high-quality publications, whereas men focused on maintaining the quality of their publications. However, in some models, gender had no significant impact on research publication quality.

Fourth, the pandemic had minimal impact on conducting research or writing grants for either group of scientists. Some quality-increasing scientists still prioritize research over grant writing, while others focus on maintaining quality and neglect grant writing.

Fifth, our analysis emphasized the differences among academic scientists in the nature of collaboration. After the pandemic, collaboration with academic scientists is crucial for maintaining the quality of research publications, while for some who aim to increase publication quality, collaboration with non-scientists is essential. Broader collaboration remains crucial for maintaining the quality of research publications.

Sixth, no single ICT tool unites academic scientists from both groups. However, the pandemic led to an increased use of ICT and social media to improve the quality of research publications. Interestingly, academic scientists who aim to increase research publication quality are less likely to use ICT than those who focus on maintaining quality. Even after the pandemic, the use of e-learning platforms remains very important, while the use of social media to maintain research publication quality continues to be limited.

Finally, academic scientists in both groups did not employ identical dissemination methods for their research findings across all research activity models. The use of print books was important for maintaining research publication quality during both periods.

Based on the results of this study, we conclude that universities can prioritize research activities and ICT tools to accelerate the identification of weaknesses, support the achievement of desired research publication quality, and stimulate the creation of individual and team synergies.

## Limitations and future research directions

The limitations of this study pertain to the following issues:

Sample size: It was challenging to recruit more participants, particularly those actively engaged in science disciplines such as Medical and Health Sciences, Agricultural Sciences, Humanities, and Natural Sciences, from South America, Antarctica, and Australia. A larger sample size would offer a clearer picture of how academic scientists work to improve or maintain the quality of research publications across various disciplines.

Dataset bias: The datasets used in this study initially exhibited highly imbalanced classes. To address this issue, we employed data processing techniques such as SMOTE and Robust Scaler to mitigate the impact of outlier values. However, these limitations within the datasets may introduce bias.

Complex datasets and the interpretability vs. accuracy trade-off in LIME: Increasing the sample size may enhance the stability of the model’s explanations by reducing uncertainty and improving the accuracy of probability estimations. Although LIME emphasizes interpretability, this focus may compromise its accuracy. Balancing interpretability and accuracy can be challenging when complex datasets are used.

Looking ahead: We recommend exploring the following fields in future research:

(i) Developing a roadmap to assess the current and future research activities of academic scientists.

(ii) Conducting a multi-criteria analysis to capture research activities related to the university’s objectives.

Further investigation: Further investigation is needed to understand the reasons for the limited use of ICTs in research activities and the neglect of certain activities. Consequently, it is valuable to explore the following questions:

(i) Do academic scientists require new ICT literacy to enhance their basic and/or applied research?

(ii) Do universities provide sufficient resources and ICT tools to support academic scientists in adapting to changes in their work?

(iii) How do ICTs impact the novelty of academic research?

(iv) Are the factors that encourage academic scientists to collaborate similar in both groups of academic scientists–those who aim to maintain quality and those who aim to increase it?

(v) Are academic scientists overwhelmed by job changes?

The study presented detailed findings on diverse models of research activities and the use of ICTs, as well as future directions. The results reveal specific values that academic scientists and universities need to reassess, leading to a new diversity in research activities and the use of ICTs that drive progress.

## Supporting information

The corpus of records from Web of Science used in the systematic literature review (S1 Table), the questionnaire (S2 Table), descriptive statistics for the data (S4 Table), and the performance comparison of five machine learning classifiers (S5 Table) can be found at: https://zenodo.org/records/10092434. The datasets corresponding to S3–S6 Tables in S3 Table, consisting of four Excel sheets, can be found at: https://zenodo.org/records/10092367.

S1 Table The corpus of records from Web of Science used in the systematic literature review. (XLS)

S2 Table The questionnaire. (DOCX)

S3 Table S3-S6 Tables. (XLSX)

S4 Table Descriptive statistics for the data. (DOCX)

S5 Table Performance comparison of five machine learning classifiers. (DOCX)

S1 Appendix (DOCX)
