# Supplementary material for: A twofold perspective on the quality of research publications: The use of ICTs and research activity models
Source: PLoS One. 2025 Jan 14;20(1):e0308952. doi: 10.1371/journal.pone.0308952 (PMC11731720; doi:10.1371/journal.pone.0308952)
Supplement: S2 Table — (DOCX) [file pone.0308952.s002.docx]

**S2 Table.** **Questionnaire research questions.**

| **Abbreviation  of variable names** | |  | | | | **Description imposed  by the 5-point Likert scale or dichotomized scale** | |
| --- | --- | --- | --- | --- | --- | --- | --- |
|  |  | **Period 1**  **During COVID-19 pandemic (D)** | | **Period 2**  **After COVID-19 pandemic (A)**  **[forecast]** | |  |  |
| D_Qa  A_Qa  (Surveys  1 and 2) | | QQ1. In your opinion, how does the quality of your research publications look like compared with the quality from before this coronavirus pandemic? [During this coronavirus pandemic] | | QQ1. In your opinion, how does the quality of your research publications change compared with the situation from before this coronavirus pandemic? [After this coronavirus pandemic (forecast)] | | Likert scale/ dichotomized scale  Definitely smaller/0  Rather smaller/0  Neither smaller nor bigger/0  Rather bigger/1  Definitely bigger/1 | |
| D_Qn  A_Qn  (Surveys  1 and 2) | | QQ2. In your opinion, how does the quantity of your research publications look like compared with the quantity from before this coronavirus pandemic? [During this coronavirus pandemic] | | QQ2. In your opinion, how does the quantity of your research publications look like compared with the quantity from before this coronavirus pandemic? [After this coronavirus pandemic (forecast)] | | Likert scale/ Dichotomized scale  Definitely smaller/0  Rather smaller/0  Neither smaller nor bigger/0  Rather bigger/1  Definitely bigger/1 | |
| D_BR  A_BR  (Surveys  1 and 2) | | QQ3. Overall, how much is your research focused on basic research in science, i.e., generating "basic" knowledge and a fundamental understanding of particular issues and topics? [During this coronavirus pandemic] | | QQ3. Overall, how much is your research focused on basic research in science, i.e., generating "basic" knowledge and a fundamental understanding of particular issues and topics? [After this coronavirus pandemic (forecast)] | | \| Never 1  Seldom 2  Sometimes 3  Often 4  Very Often 5 \| \| --- \| | |
| D_AR  A_AR  (Surveys  1 and 2) | | QQ4. Overall, how much is your research focused on applied research and research for development, i.e., practical implications and immediate, potential uses and applications? (e.g., commercial potential, policy relevance) [During this coronavirus pandemic] | | QQ4. Overall, how much is your research focused on applied research and research for development, i.e., practical implications and immediate, potential uses and applications?(e.g., commercial potential, policy relevance) [After this coronavirus pandemic (forecast)] | | Never 1  Seldom 2  Sometimes 3  Often 4  Very Often 5 | |
| D_Research  A_Research  (Surveys  1 and 2) | | QQ5. How do you spend your work hours during this coronavirus pandemic? Please rate the following areas in order from the one you spend the most time on, to the one you spend the least time on. (Please move the blocks from the left column to the right column by drag-and-drop or double click on each block) [During this coronavirus pandemic] | | QQ5. In your opinion, how will you spend your work hours after this coronavirus pandemic? (forecast)Please rate following areas in order from the one you spend the most time on, to the one you spend the least time on. (Please move the blocks from the left column to the right column by drag-and-drop or double click on each block) | | \| Rank 1 - First place  Rank 2 - Second place  Rank 3 - Third place  Rank 4 - Last place \| \| --- \| | |
| D_Grant_wr  A_Grant_wr  (Surveys  1 and 2) | | QQ6. How do you spend your work hours during this coronavirus pandemic? Please rate the following areas in order from the one you spend the most time on, to the one you spend the least time on. (Please move the blocks from the left column to the right column by drag-and-drop or double click on each block) [During this coronavirus pandemic] | | QQ6. In your opinion, how will you spend your work hours after this coronavirus pandemic? (forecast)Please rate following areas in order from the one you spend the most time on, to the one you spend the least time on. (Please move the blocks from the left column to the right column by drag-and-drop or double click on each block) | | \| Rank 1 - First place  Rank 2 - Second place  Rank 3 - Third place  Rank 4 - Last place \| \| --- \| | |
| D_Com_apps  A_Com_apps  (Surveys  1 and 2) | | QQ7. How do you assess the frequency of usage of any communication apps in your research? (e.g., Skype, WhatsApp, Google Meet, Messenger, MS Teams) [During this coronavirus pandemic] | | QQ7. How do you assess the frequency of usage of any communication apps in your research? (e.g., Skype, WhatsApp, Google Meet, Messenger, MS Teams)  [After this coronavirus pandemic (forecast)] | | Never 1  Seldom 2  Sometimes 3  Often 4  Very Often 5 | |
| D_E_learn_platf  A_E_learn_platf  (Surveys  1 and 2) | | QQ8. How do you assess the frequency of usage of any e-learning platforms in your research? (e.g., Moodle; Google Classroom; Zoom; Docebo; Wiz IQ;  ATutor; MS Teams)  [During this coronavirus pandemic] | | QQ8. How do you assess the frequency of usage of any e-learning platforms in your research? (e.g., Moodle; Google Classroom; Zoom; Docebo; Wiz IQ;  ATutor; MS Teams)  [After this coronavirus pandemic (forecast)] | | Never 1  Seldom 2  Sometimes 3  Often 4  Very Often 5 | |
| D_Online_conf  A_Online_conf  (Surveys  1 and 2) | | QQ9. In your opinion, for the research projects you participate in during this coronavirus pandemic, please rate the importance of the following inputs [Attendance at online conferences, webinars etc.] [During this coronavirus pandemic] | | QQ9. In your opinion, for the research projects that you will participate in after this coronavirus pandemic, please rate the importance of the following inputs (forecast) [Attendance at online conferences, webinars etc.] | | Definitely unimportant 1  Rather unimportant 2  Neither important nor unimportant 3  Rather important 4  Definitely important 5 | |
| D_Trad_conf  A_Trad_conf  (Surveys  1 and 2) | | QQ10. In your opinion, for the research projects you participate in during this coronavirus pandemic, please rate the importance of the following inputs [Attendance at traditional conferences, seminars]  [During this coronavirus pandemic] | | QQ10. In your opinion, for the research projects that you will participate in after this coronavirus pandemic, please rate the importance of the following inputs (forecast) [Attendance at traditional conferences, seminars] | | Definitely unimportant 1  Rather unimportant 2  Neither important nor unimportant 3  Rather important 4  Definitely important 5 | |
| D_Social_media  A_Social_media  (Surveys  1 and 2) | | QQ11. How do you assess the frequency of usage of any social media in your research? (e.g., Facebook, Twitter, LinkedIn, Youtube, Instagram, blog sites) [During this coronavirus pandemic] | | QQ11. How do you assess the frequency of usage of any social media in your research? (e.g., Facebook, Twitter, LinkedIn, Youtube, Instagram, blog sites) [After this coronavirus pandemic (forecast)] | | Never 1  Seldom 2  Sometimes 3  Often 4  Very Often 5 | |
| D_Stat_softw  A_Stat_softw  (Survey 1) | | QQ12. How do you assess the frequency of usage of any statistical analysis software in your research? (e.g., MATLab; Statistica; SPSS; SageMath) [During this coronavirus pandemic] | | QQ12. How do you assess the frequency of usage of any statistical analysis software in your research? (e.g., MATLab; Statistica; SPSS; SageMath) [After this coronavirus pandemic (forecast)] | | Never 1  Seldom 2  Sometimes 3  Often 4  Very Often 5 | |
| D_Qu_softw  A_Qu_softw  (Survey 1) | | QQ13. How do you assess the frequency of usage of any questionnaire software in your research? (e.g., Candel; Charted; Datawrapper; Leaflet; LimeSurvey; SurveyMonkey) [During this coronavirus pandemic] | | QQ13. How do you assess the frequency of usage of any questionnaire software in your research? (e.g., Candel; Charted; Datawrapper; Leaflet; LimeSurvey; SurveyMonkey) [After this coronavirus pandemic (forecast)] | | Never 1  Seldom 2  Sometimes 3  Often 4  Very Often 5 | |
| D_E_ journ  A_E_ journ  (Survey 1) | | QQ14. How do you assess the frequency of usage of the following resources in your research during this coronavirus pandemic? [E-journals] | | QQ14. How do you assess the frequency of usage of the following resources in your research after this coronavirus pandemic? (forecast) [E-journals] | | Never 1  Seldom 2  Sometimes 3  Often 4  Very Often 5 | |
| D_Print_journ  A_Print_journ  (Survey 1) | | QQ15. How do you assess the frequency of usage of the following resources in your research during this coronavirus pandemic? (forecast) [Journals (print copies) | | QQ15. How do you assess the frequency of usage of the following resources in your research after this coronavirus pandemic? (forecast) [Journals (print copies)] | | Never 1  Seldom 2  Sometimes 3  Often 4  Very Often 5 | |
| D_E_book  A_E_book  (Survey 1) | | QQ16. How do you assess the frequency of usage of the following resources in your research during this coronavirus pandemic? [Ebooks] | | QQ16. How do you assess the frequency of usage of the following resources in your research after this coronavirus pandemic? (forecast) [Ebooks] | | Never 1  Seldom 2  Sometimes 3  Often 4  Very Often 5 | |
| D_Print_book  A_Print_book  (Survey 1) | | QQ17. How do you assess the frequency of usage of the following resources in your research during this coronavirus pandemic? [Books (paper editions)] | | QQ17. How do you assess the frequency of usage of the following resources in your research after this coronavirus pandemic? (forecast) [Books (paper editions)] | | Never 1  Seldom 2  Sometimes 3  Often 4  Very Often 5 | |
| D_Online_db  A_Online_db  (Survey 1) | | QQ18. How do you assess the frequency of usage of the following resources in your research during this coronavirus pandemic? [Online databases] | | QQ18. How do you assess the frequency of usage of the following resources in your research after this coronavirus pandemic? (forecast) [Online databases] | | Never 1  Seldom 2  Sometimes 3  Often 4  Very Often 5 | |
| D_Collab(foreign_r)  A_Collab(foreign_r)  (Survey 1) | | QQ19. In your opinion, for the research projects you participate in during this coronavirus pandemic, please rate the importance of the following inputs [Collaboration with foreign researchers] | | QQ19. In your opinion, for the research projects that you will participate in after this coronavirus pandemic, please rate the importance of the following inputs (forecast) [Collaboration with foreign researchers] | | Definitely unimportant 1  Rather unimportant 2  Neither important nor unimportant 3  Rather important 4  Definitely important 5 | |
| D_Collab (uni_faculty)  A_Collab( uni_faculty)  (Survey 1) | | QQ20. For the research projects you participate in during this coronavirus pandemic, please rate the importance of the following inputs [Collaboration with your university or faculty’s researchers] | | QQ20. For the research projects that you will participate in after this coronavirus pandemic, please rate the importance of the following inputs (forecast) [Collaboration with your university or faculty’s researchers] | | \| Definitely unimportant 1  Rather unimportant 2  Neither important  nor unimportant 3  Rather important 4  Definitely important 5 \| 1 \| \| --- \| --- \| \| 2 \| \| 3 \| \| 4 \| \| 5 \| | |
| D_Collab (postdoc_st)  A_Collab (postdoc_st)  (Survey 1) | | QQ21. For the research projects you participate in during this coronavirus pandemic, please rate the importance of the following inputs [Collaboration with post-docs, grad students] | | QQ21. For the research projects that you will participate in after this coronavirus pandemic, please rate the importance of the following inputs (forecast) [Collaboration with post-docs, grad students] | | Definitely unimportant 1  Rather unimportant 2  Neither important  nor unimportant 3  Rather important 4  Definitely important 5 | |
| D_Collab(outside_uni)  A_Collab(outside_uni)  (Survey 1) | | QQ22. For the research projects you participate in during this coronavirus pandemic, please rate the importance of the following inputs [Collaboration with domestic researchers from outside your university] | | QQ22. For the research projects that you will participate in after this coronavirus pandemic, please rate the importance of the following inputs (forecast) [Collaboration with domestic researchers from outside your university] | | Definitely unimportant 1  Rather unimportant 2  Neither important  nor unimportant 3  Rather important 4  Definitely important 5 | |
| D_Collab( w_others)  A_Collab (w_others)  (Survey 1) | | QQ23. For the research projects you participate in during this coronavirus pandemic, please rate the importance of the following inputs [Collaboration with others] | | QQ23. For the research projects that you will participate in after this coronavirus pandemic, please rate the importance of the following inputs (forecast) [Collaboration with others] | | Definitely unimportant 1  Rather unimportant 2  Neither important  nor unimportant 3  Rather important 4  Definitely important 5 | |
|  | | Demographic characteristics, science discipline and teaching model | | | |  | |
| Age  (Surveys  1 and 2) | | \| QQ24. Please specify your age \| \| --- \| | | | | \| < 20 1  20 – 34 2  35 – 49 3  50 – 68 4  >69 5 \| 1 \| \| --- \| --- \| \| 2 \| \| 3 \| \| 4 \| \| 5 \| \| 6 \| | |
| Gender  (Surveys  1 and 2) | | QQ25. Please specify your gender | | | | Female 1  Male 2 | |
| PL_Aborad  (Surveys  1 and 2) | | QQ26. Enter the country in which you work | | | | Dichotomized scale  Poland 1  Abroad 2 | |
| OECD  (Surveys  1 and 2) | | QQ27. Which of the following field of science and technology best describes your research?  (OECD Classification http://www.oecd.org/science/inno/38235147.pdf) | | | | \| Social sciences 1  Engineering and technology 2  Humanities 3  Natural sciences 4  Medical and health sciences 5  Agricultural sciences 6 \| 1 \| \| --- \| --- \| \| 2 \| \| 3 \| \| 4 \| \| 5 \| \| 6 \| | |
| Scientific_ position  (Surveys  1 and 2) | | QQ28. Which of the following best describes your current scientific position? | | | | Ph.D.-student 1  Lecturer 2  Assistant Professor 3  Associate Professor 4  Professor 5  Retired 6  Researcher 7  Assistant 8  Other 9 | |
| D_Teaching_ model  A_Teaching_ model  (Surveys  1 and 2) | | QQ29. Which of the following teaching model best describes your university? [During this coronavirus pandemic] | | QQ24. Which of the following teaching model best describes your university? [After this coronavirus pandemic (forecast)] | | \| Traditional 1  Traditional + online 2  Online 3 \| 1 \| \| --- \| --- \| \| 2 \| \| 3 \| | |

Source: Authors’ elaboration based on [Ziemba, E. W., Maruszewska, E. W., Eisenbardt, M., Grabara, D., Wartini-Twardowska, J., & Tuszkiewicz, M. (2023). Research Survey on 'Effects of the Coronavirus Pandemic on Scientific Research' [Data set]. Zenodo. https://doi.org/10.5281/zenodo.10092367**].**
