# Supplementary material for: A twofold perspective on the quality of research publications: The use of ICTs and research activity models
Source: PLoS One. 2025 Jan 14;20(1):e0308952. doi: 10.1371/journal.pone.0308952 (PMC11731720; doi:10.1371/journal.pone.0308952)
Supplement: S4 Table — (DOCX) [file pone.0308952.s004.docx]

**S7 Table. Descriptive statistics for research activities and ICT used in academic research to increase or maintain the quality of research publications.**

|  | | **Surveys 1 and 2 (Period 1)** | | | | | | | | | | | | | | | | | |  | | |  | | | | **Surveys 1 and 2 (Period 2)** | | | | | | | | | | | | | | | | | |  |  |  |
| --- | --- | --- | --- | --- | --- | --- | --- | --- | --- | --- | --- | --- | --- | --- | --- | --- | --- | --- | --- | --- | --- | --- | --- | --- | --- | --- | --- | --- | --- | --- | --- | --- | --- | --- | --- | --- | --- | --- | --- | --- | --- | --- | --- | --- | --- | --- | --- |
| Index | | count | mean | | std | min | | | 25% | | 50% | | 75% | | max | | | mode |  | | | Index | | | | count | | | | mean | | std | min | | | 25% | | 50% | | 75% | | max | | mode | | |  |
| D_Qa | | 476.0 | 0.2 | | 0.4 | 0.0 | | | 0.0 | | 0.0 | | 0.0 | | 1.0 | | | 0.0 |  | | | A_Qa | | | | 454.0 | | | | 0.3 | | 0.5 | 0.0 | | | 0.0 | | 0.0 | | 1.0 | | 1.0 | | 0.0 | | |  |
| D_Qn | | 476.0 | 0.2 | | 0.4 | 0.0 | | | 0.0 | | 0.0 | | 0.0 | | 1.0 | | | 0.0 |  | | | A_Qn | | | | 454.0 | | | | 0.3 | | 0.5 | 0.0 | | | 0.0 | | 0.0 | | 1.0 | | 1.0 | | 0.0 | | |  |
| D_BR | | 476.0 | 3.2 | | 1.1 | 1.0 | | | 2.0 | | 3.0 | | 4.0 | | 5.0 | | | 4.0 |  | | | A_BR | | | | 454.0 | | | | 3.4 | | 1.0 | 1.0 | | | 3.0 | | 3.0 | | 4.0 | | 5.0 | | 4.0 | | |  |
| D_AR | | 476.0 | 3.4 | | 1.2 | 1.0 | | | 3.0 | | 4.0 | | 4.0 | | 5.0 | | | 4.0 |  | | | A_AR | | | | 454.0 | | | | 3.7 | | 1.0 | 1.0 | | | 3.0 | | 4.0 | | 4.0 | | 5.0 | | 4.0 | | |  |
| D_Research | | 476.0 | 2.0 | | 0.9 | 1.0 | | | 1.0 | | 2.0 | | 3.0 | | 4.0 | | | 1.0 |  | | | A_Research | | | | 454.0 | | | | 1.6 | | 0.8 | 1.0 | | | 1.0 | | 1.0 | | 2.0 | | 4.0 | | 1.0 | | |  |
| D_Grant_wr | | 476.0 | 3.5 | | 0.8 | 1.0 | | | 3.0 | | 4.0 | | 4.0 | | 4.0 | | | 4.0 |  | | | A_Grant_wr | | | | 454.0 | | | | 3.4 | | 0.8 | 1.0 | | | 3.0 | | 4.0 | | 4.0 | | 4.0 | | 4.0 | | |  |
| D_Com_apps | | 476.0 | 4.0 | | 1.2 | 1.0 | | | 3.0 | | 4.0 | | 5.0 | | 5.0 | | | 5.0 |  | | | A_Com_apps | | | | 454.0 | | | | 3.7 | | 1.0 | 1.0 | | | 3.0 | | 4.0 | | 4.0 | | 5.0 | | 4.0 | | |  |
| D_E_learn_platf | | 476.0 | 3.7 | | 1.5 | 1.0 | | | 3.0 | | 4.0 | | 5.0 | | 5.0 | | | 5.0 |  | | | A_E_learn_platf | | | | 454.0 | | | | 3.5 | | 1.2 | 1.0 | | | 3.0 | | 4.0 | | 4.0 | | 5.0 | | 4.0 | | |  |
| D_Online_conf | | 476.0 | 3.9 | | 1.1 | 1.0 | | | 3.8 | | 4.0 | | 5.0 | | 5.0 | | | 4.0 |  | | | A_Online_conf | | | | 454.0 | | | | 3.9 | | 0.8 | 1.0 | | | 3.0 | | 4.0 | | 4.0 | | 5.0 | | 4.0 | | |  |
| D_Trad_conf | | 476.0 | 2.4 | | 1.3 | 1.0 | | | 1.0 | | 2.0 | | 3.0 | | 5.0 | | | 1.0 |  | | | A_Trad_conf | | | | 454.0 | | | | 3.8 | | 0.9 | 1.0 | | | 3.0 | | 4.0 | | 4.0 | | 5.0 | | 4.0 | | | |
| D_Social_media | | 476.0 | 2.6 | | 1.4 | 1.0 | | | 1.0 | | 3.0 | | 4.0 | | 5.0 | | | 1.0 |  | | | A_Social_media | | | | 454.0 | | | | 2.6 | | 1.3 | 1.0 | | | 1.0 | | 3.0 | | 4.0 | | 5.0 | | 1.0 | | |  |
|  | | **Survey 1 (Period 1)** | | | | | | | | | | | | | | | | | |  | | |  |  | | | | **Survey 1 (Period 2)** | | | | | | | | | | | | | | | | | | | |
| Index | | count | mean | | | std | min | | 25% | | 50% | | 75% | | max | mode | | | |  | | | Index | | | | count | | | mean | | | std | min | | 25% | | 50% | | 75% | | max | | | mode | | |
| D_Qa | | 152.0 | 0.2 | | | 0.4 | 0.0 | | 0.0 | | 0.0 | | 0.0 | | 1.0 | 0.0 | | | |  | | | A_Qa | | | | 152.0 | | | 0.3 | | | 0.4 | 0.0 | | 0.0 | | 0.0 | | 1.0 | | 1.0 | | | 0.0 | | |
| D_Qn | | 152.0 | 0.2 | | | 0.4 | 0.0 | | 0.0 | | 0.0 | | 0.0 | | 1.0 | 0.0 | | | |  | | | A_Qn | | | | 152.0 | | | 0.3 | | | 0.4 | 0.0 | | 0.0 | | 0.0 | | 1.0 | | 1.0 | | | 0.0 | | |
| D_BR | | 152.0 | 3.1 | | | 1.2 | 1.0 | | 2.0 | | 3.0 | | 4.0 | | 5.0 | 4.0 | | | |  | | | A_BR | | | | 152.0 | | | 3.3 | | | 1.1 | 1.0 | | 2.0 | | 3.0 | | 4.0 | | 5.0 | | | 4.0 | | |
| D_AR | | 152.0 | 3.4 | | | 1.2 | 1.0 | | 3.0 | | 4.0 | | 4.0 | | 5.0 | 4.0 | | | |  | | | A_AR | | | | 152.0 | | | 3.8 | | | 1.0 | 1.0 | | 3.0 | | 4.0 | | 4.2 | | 5.0 | | | 4.0 | | |
| D_Research | | 152.0 | 1.9 | | | 0.9 | 1.0 | | 1.0 | | 2.0 | | 3.0 | | 4.0 | 1.0 | | | |  | | | A_Research | | | | 152.0 | | | 1.6 | | | 0.7 | 1.0 | | 1.0 | | 1.0 | | 2.0 | | 4.0 | | | 1.0 | | |
| D_Grant_wr | | 152.0 | 3.6 | | | 0.8 | 1.0 | | 3.0 | | 4.0 | | 4.0 | | 4.0 | 4.0 | | | |  | | | A_Grant_wr | | | | 152.0 | | | 3.4 | | | 0.8 | 1.0 | | 3.0 | | 4.0 | | 4.0 | | 4.0 | | | 4.0 | | |
| D_Com_apps | | 152.0 | 4.4 | | | 1.0 | 1.0 | | 4.0 | | 5.0 | | 5.0 | | 5.0 | 5.0 | | | |  | | | A_Com_apps | | | | 152.0 | | | 3.9 | | | 0.9 | 1.0 | | 3.0 | | 4.0 | | 5.0 | | 5.0 | | | 4.0 | | |
| D_E_learn_platf | | 152.0 | 4.3 | | | 1.1 | 1.0 | | 4.0 | | 5.0 | | 5.0 | | 5.0 | 5.0 | | | |  | | | A_E_learn_platf | | | | 152.0 | | | 3.8 | | | 1.1 | 1.0 | | 3.0 | | 4.0 | | 5.0 | | 5.0 | | | 4.0 | | |
| D_Online_conf | | 152.0 | 3.9 | | | 1.1 | 1.0 | | 3.0 | | 4.0 | | 5.0 | | 5.0 | 5.0 | | | |  | | | A_Online_conf | | | | 152.0 | | | 3.8 | | | 0.9 | 1.0 | | 3.0 | | 4.0 | | 4.0 | | 5.0 | | | 4.0 | | |
| D_Trad_conf | | 152.0 | 2.4 | | | 1.2 | 1.0 | | 1.0 | | 2.0 | | 3.0 | | 5.0 | 4.0 | | | |  | | | A_Trad_conf | | | | 152.0 | | | 3.7 | | | 1.0 | 1.0 | | 3.0 | | 4.0 | | 4.0 | | 5.0 | | | 4.0 | | |
| D_Social_media | | 152.0 | 2.7 | | | 1.5 | 1.0 | | 1.0 | | 3.0 | | 4.0 | | 5.0 | 1.0 | | | |  | | | A_Social_media | | | | 152.0 | | | 2.8 | | | 1.4 | 1.0 | | 1.0 | | 3.0 | | 4.0 | | 5.0 | | | 1.0 | | |

|  | | | **Survey 1 (Period 1)** | | | | | | | | | | | | | | | | | | | |  | |  |  | | | **Survey 1 (Period 2)** | | | | | | | | | | | | | | | | |  |  |  |
| --- | --- | --- | --- | --- | --- | --- | --- | --- | --- | --- | --- | --- | --- | --- | --- | --- | --- | --- | --- | --- | --- | --- | --- | --- | --- | --- | --- | --- | --- | --- | --- | --- | --- | --- | --- | --- | --- | --- | --- | --- | --- | --- | --- | --- | --- | --- | --- | --- |
| Index | count | | | | mean | | std | | min | | 25% | | 50% | | 75% | | max | | mode | |  | | | Index | | | count | | | mean | | std | | min | | 25% | | 50% | | 75% | | max | | mode | | |  |  |
| D_Stat_softw | | | 152.0 | | 3.4 | | 1.4 | | 1.0 | | 2.0 | | 4.0 | | 5.0 | | 5.0 | | 4.0 | |  | | A_Stat_softw | | | | | | 152.0 | | | 3.5 | | 1.3 | | 1.0 | | 3.0 | | 4.0 | | 5.0 | | 5.0 | | 4.0 | | |
| D_Qu_softw | | | 152.0 | | 2.8 | | 1.4 | | 1.0 | | 1.0 | | 3.0 | | 4.0 | | 5.0 | | 1.0 | |  | | A_Qu_softw | | | | | | 152.0 | | | 2.9 | | 1.3 | | 1.0 | | 2.0 | | 3.0 | | 4.0 | | 5.0 | | 4.0 | | |
| D_E_journ | | | 152.0 | | 4.4 | | 0.8 | | 1.0 | | 4.0 | | 5.0 | | 5.0 | | 5.0 | | 5.0 | |  | | A_E_journ | | | | | | 152.0 | | | 4.4 | | 0.7 | | 1.0 | | 4.0 | | 5.0 | | 5.0 | | 5.0 | | 5.0 | | |
| D_Print_journ | | | 152.0 | | 2.5 | | 1.2 | | 1.0 | | 1.0 | | 2.0 | | 3.2 | | 5.0 | | 2.0 | |  | | A_Print_journ | | | | | | 152.0 | | | 3.2 | | 1.1 | | 1.0 | | 2.0 | | 3.0 | | 4.0 | | 5.0 | | 4.0 | | |
| D_E_book | | | 152.0 | | 4.0 | | 1.0 | | 1.0 | | 3.8 | | 4.0 | | 5.0 | | 5.0 | | 4.0 | |  | | A_E_book | | | | | | 152.0 | | | 4.2 | | 0.8 | | 1.0 | | 4.0 | | 4.0 | | 5.0 | | 5.0 | | 4.0 | | |
| D_Print_book | | | 152.0 | | 3.0 | | 1.2 | | 1.0 | | 2.0 | | 3.0 | | 4.0 | | 5.0 | | 4.0 | |  | | A_Print_book | | | | | | 152.0 | | | 3.5 | | 1.1 | | 1.0 | | 3.0 | | 4.0 | | 4.0 | | 5.0 | | 4.0 | | |
| D_Online_db | | | 152.0 | | 4.2 | | 0.9 | | 1.0 | | 4.0 | | 4.0 | | 5.0 | | 5.0 | | 5.0 | |  | | A_Online_db | | | | | | 152.0 | | | 4.4 | | 0.8 | | 1.0 | | 4.0 | | 5.0 | | 5.0 | | 5.0 | | 5.0 | | |
| D_Collab(foreign_r) | | | 152.0 | | 3.5 | | 1.1 | | 1.0 | | 3.0 | | 4.0 | | 4.0 | | 5.0 | | 4.0 | |  | | A_Collab(foreign_r) | | | | | | 152.0 | | | 2.9 | | 1.4 | | 1.0 | | 1.0 | | 4.0 | | 4.0 | | 5.0 | | 4.0 | | |
| D_Collab(uni_faculty) | | | 152.0 | | 3.7 | | 1.1 | | 1.0 | | 3.0 | | 4.0 | | 5.0 | | 5.0 | | 4.0 | |  | | A_Collab(uni_faculty) | | | | | | 152.0 | | | 2.8 | | 1.4 | | 1.0 | | 1.0 | | 3.0 | | 4.0 | | 5.0 | | 4.0 | | |
| D_Collab(postdoc_st) | | | 152.0 | | 3.0 | | 1.2 | | 1.0 | | 2.0 | | 3.0 | | 4.0 | | 5.0 | | 3.0 | |  | | A_Collab(postdoc_st) | | | | | | 152.0 | | | 3.3 | | 1.1 | | 1.0 | | 3.0 | | 3.5 | | 4.0 | | 5.0 | | 4.0 | | |
| D_Collab(r_outside_uni) | | | 152.0 | | 3.5 | | 1.1 | | 1.0 | | 3.0 | | 4.0 | | 4.0 | | 5.0 | | 4.0 | |  | | A_Collab(r_outside_uni) | | | | | | 152.0 | | | 3.2 | | 1.3 | | 1.0 | | 2.0 | | 4.0 | | 4.0 | | 5.0 | | 4.0 | | |
| D_Collab(w_others) | | | 152.0 | | 3.3 | | 1.0 | | 1.0 | | 3.0 | | 4.0 | | 4.0 | | 5.0 | | 4.0 | |  | | A_Collab(w_others) | | | | | | 152.0 | | | 3.0 | | 1.1 | | 1.0 | | 2.0 | | 3.0 | | 4.0 | | 5.0 | | 4.0 | | |
