# Supplementary material for: A twofold perspective on the quality of research publications: The use of ICTs and research activity models
Source: PLoS One. 2025 Jan 14;20(1):e0308952. doi: 10.1371/journal.pone.0308952 (PMC11731720; doi:10.1371/journal.pone.0308952)
Supplement: S5 Table — (DOCX) [file pone.0308952.s005.docx]

**S8 Table. Performance comparison of five machine learning classifiers for the quality of research publications and its explanatory variables using SMOTE and cross-validation = 10.**

| **Machine learning classifier** | **Survey and period** | **Mean balanced accuracy** | | **ROC_AUC train** | **ROC_AUC test** | **F1-score** | **G-mean** |
| --- | --- | --- | --- | --- | --- | --- | --- |
|  |  | **Training dataset** | **Testing dataset** | **Training dataset** | **Testing dataset** | **Testing dataset** | **Testing dataset** |
| LinearDiscriminantAnalysis | Surveys 1 and 2 (Period 1) | 0.79 | 0.82 | 0.87 | 0.82 | 0.87 | 0.82 |
| LinearDiscriminantAnalysis | Surveys 1 and 2 (Period 2) | 0.77 | 0.81 | 0.81 | 0.88 | 0.84 | 0.81 |
| LinearDiscriminantAnalysis | Survey 1 (Period 1) | 0.81 | 0.88 | 0.88 | 0.86 | 0.94 | 0.88 |
| LinearDiscriminantAnalysis | Survey 1 (Period 2) | 0.71 | 0.79 | 0.77 | 0.79 | 0.86 | 0.77 |
| LogisticRegression | Surveys 1 and 2 (Period 1) | 0.80 | 0.81 | 0.88 | 0.88 | 0.86 | 0.81 |
| LogisticRegression | Surveys 1 and 2 (Period 2) | 0.76 | 0.82 | 0.80 | 0.87 | 0.84 | 0.82 |
| LogisticRegression | Survey 1 (Period 1) | 0.82 | 0.76 | 0.92 | 0.79 | 0.87 | 0.74 |
| LogisticRegression | Survey 1 (Period 2) | 0.73 | 0.81 | 0.79 | 0.82 | 0.84 | 0.81 |
| LinearSVC | Surveys 1 and 2 (Period 1) | 0.77 | 0.81 | 0.85 | 0.92 | 0.86 | 0.81 |
| LinearSVC | Surveys 1 and 2 (Period 2) | 0.77 | 0.84 | 0.79 | 0.88 | 0.86 | 0.83 |
| LinearSVC | Survey 1 (Period 1) | 0.73 | 0.78 | 0.85 | 0.79 | 0.90 | 0.76 |
| LinearSVC | Survey 1 (Period 1) | 0.66 | 0.73 | 0.72 | 0.73 | 0.83 | 0.69 |
| BaggingClassifier | Surveys 1 and 2 (Period 1) | 0.72 | 0.67 | 0.76 | 0.70 | 0.79 | 0.65 |
| BaggingClassifier | Surveys 1 and 2 (Period 2) | 0.63 | 0.64 | 0.69 | 0.69 | 0.70 | 0.63 |
| BaggingClassifier | Survey 1 (Period 1) | 0.76 | 0.68 | 0.78 | 0.62 | 0.86 | 0.62 |
| BaggingClassifier | Survey 1 (Period 2) | 0.62 | 0.70 | 0.67 | 0.75 | 0.70 | 0.70 |
| ExtraTreeClassifier | Surveys 1 and 2 (Period 1) | 0.78 | 0.80 | 0.87 | 0.86 | 0.88 | 0.79 |
| ExtraTreesClassifier | Surveys 1 and 2 (Period 2) | 0.76 | 0.74 | 0.81 | 0.86 | 0.79 | 0.74 |
| ExtraTreeClassifier | Survey 1 (Period 1) | 0.72 | 0.9 | 0.89 | 0.83 | 0.97 | 0.89 |
| ExtraTreeClassifier | Survey 1 (Period 2) | 0.71 | 0.69 | 0.78 | 0.84 | 0.81 | 0.61 |
