# Supplementary material for: A twofold perspective on the quality of research publications: The use of ICTs and research activity models
Source: PLoS One. 2025 Jan 14;20(1):e0308952. doi: 10.1371/journal.pone.0308952 (PMC11731720; doi:10.1371/journal.pone.0308952)
Supplement: S1 Appendix — (DOCX) [file pone.0308952.s006.docx]

**Appendix 1. Synthesis of the systematic literature review on academic scientists’ research activities and ICT use in research.**

| **Author  (year)** | **Research method** | **Research focus** | **Selected findings** |
| --- | --- | --- | --- |
| **1** | **2** | **4** | **5** |
| Abramo  and  D’Angelo (2023) [31] | Bibliometric approach; The Web of Science database; Interrupted Time Series; Italian research assessment and top 15 countries with significant scientific output over 14 years; N ranges from 1,260,000 in 2006 to 2,130,000 in 2019. | The intensity of international research collaboration | - The rate of internationalization for researchers of Italian national institutions has doubled compared to global counterparts, resulting in increased international research collaboration. |
| Ahmad et al. (2022) [32] | Elsevier’s SciVal and Web of Science’s InCites; 2009–2018 period; The non-parametric Spearman correlation coefficient; N=134 Pakistani elite researchers. | Determinants of productivity and impact | - Elite researchers prioritize international and industry collaborations, as well as publishing in top journals with impact factor/citation score and open access. |
| Aiyebelehin (2022) [33] | Case study; The universe collaborative model as basis for narrative; Nigerian academic scientists. | The “add-my-name” as a parody of research collaboration | - Nigerian researchers often seek authorship credit on articles without contributing intellectually. |
| Amarante  and Zurbrigg (2022) [34] | Bibliometric approach; The Scopus database; 1990-2019 period; The top 20 development journals based on  “h5-index;” A regression: a probit model and a linear probability model; Multinomial logistic model; N =23713 articles. | Marginalization of southern academics and their research | - Research from the Northern Hemisphere is cited more frequently than research from developing countries. - The composition of the editorial board is strongly correlated with the origin of the publications. - Collaborations between researchers from northern and southern hemispheres have significantly increased. |
| Azmeh (2022) [2] | Bibliometric approach; The Scopus database; 2000–17 period; 27 subject fields; 15 Middle East & North Africa countries (MENA); The dynamic panel Generalized Method of Moments estimation. | The relationship between quality, quantity of research and economic growth | - The quality of research has a positive impact, and quantity has a negative impact on economic growth in almost all fields of research in MENA countries. |
| Cho et al. (2022) [35] | Bibliometric approach; Seven journals; Citation and journal data; A principal component analysis; N = the 50 most cited accounting authors. | The impact of environmental accounting research | - Top accounting authors publish in non-top-quality journals. Environment-themed journals are disadvantaged in rankings. |
| Gopalakrishna  et al. (2022) [3] | Online survey; 75 questions; Multiple (i) linear regression, (ii) binary logistic regression and (iii) ordinal logistic regression; N = 6 813 academic researchers from the Netherlands. | Occurrences of questionable research practices (QRPs) and research misconduct (FFs) | - PhD candidates, junior researchers, and male researchers are more likely to engage in QRPs due to the pressure of publishing research. Fabrication is more common than falsification in academic research practices. - Most individuals engage in at least one QRP. |
| Hou et al. (2022) [36] | Bibliometric approach; List of journals based on the Journal Citation Reports by Clarivate Analytics and Scopus database for the period 1971-2019; A field of business; Correlation coefficients; Poisson regression; N = 1,933 scholars and 88,875 articles. | Predictor of long-term scientific success | - Researchers who publish in top journals early in their careers perform better compared to peers with similar early career profiles who do not have publications in top journals. |
| Huang and Yang (2022) [8] | Bibliometric approach; The Web of Science Core Collection database; 2017–18 period; Citations received in 2019; Journals under the category field of Ecology; Spearman and Pearson correlations; Cumulative percentage; N=34 164 journal articles in 157 journals. | Quantifying journal performance | - Top academic articles can be better identified using extended metrics, such as superior identification efficiency and article rank percentile. |
| Nicholas et al. (2022) [37] | Mixed approach; Three interviews in 2021 twice; Follow-up questionnaire survey; Descriptive statistics; N = 177 early career researchers (ECRs) from eight countries. | Factors of choosing the right journal | - Journal quality is crucial for ECRs and is measured by factors such as prestige, impact factor, and database indexation. |
| Sarkar et al. (2022) [38] | Bibliometric approach; The Web of Science database; Metadata of articles published by universities in the domain of Computer Science; 2012-13 period; The Constant Elasticity of Substitution function; N=75 universities from The Times Higher Education Rankings. | Calculation of the universities’ scores | - Score calculation proposal for smaller institutions using the global influence of citations and international collaboration ratio. |
| Siler et al. (2022) [39] | Bibliometric approach; The Clarivate Analytics Web of Science; 1980–2017 period; The crossed mixed-effect logistic regression; N=347 economics journals and three major generalist science journals | Cumulative advantage versus citation counts | - Authorship repetition in top economics journals lowers citation counts, especially for debut articles. |
| Song et al. (2022) [6] | Three experiments in three different periods; The Muenster Epistemic Trustworthiness Inventory; Different scales: 5-point Likert scale; A 7-point scale; A 101-point feeling thermometer; Cohen’s d using 10,000 percentile-based bootstrap replicates; A one-way ANOVA; N=2,691 the general public and academics. | Trust for open science | - The public and academics view open science research more positively than non-open science research. |
| Akbaritabar et al. (2021) [40] | Bibliometric approach; The Scopus database - journals and book series; 2006-15 period; The MIUR website; Italian sociologists; Distributions; Bayesian models; N = n.d. | The impact of a national research assessment on the publications | - Adaptive responses can be stimulated by strategic journal targeting for publication. |
| Anzt et al. (2021) [41] | Case study | Open-source software  as a contribution to research | - Submitting open-source software as conference contributions promotes development, advances the scientific community, and enhances the capabilities of software systems. |
| Brzica (2021) [42] | Bibliometric approach. The Web of Science database; January 1, 2008 – April 30 2018; The correlation coefficient; Regression analysis - linear model; N= 88 653 individuals. | Publication and citation patterns | - Highly productive global researchers have more citations but lower citations per publication than the average. |
| Chen et al. (2021) [43] | The Kuosmanen and Mation’s integer DEA model; A non-parametric method to measure the relative efficiency of decision-making units; Management science and operations research field. | Approach for academic journal assessment | - The enhanced Integer Data Envelopment Analysis (DEA) model can identify the most significant journals. - DEA scores should be used as ranking scores. |
| Craig et al. (2021) [5] | Elsevier’s SciVal; The Scopus Database; Three-week study starting in late January 2020;  Kruskal–Wallis H tests; Dunn’s post-hoc tests with Bonferroni corrected p-values; Robust regression; N= 749 Australian psychology academics. | Research productivity, quality, and impact metrics | - Publishing in top journals does not always mean more citations. - Short-term measuring using international collaboration and SciVal citations can predict future research productivity. |
| D'Ippoliti (2021) [44] | Quantitative study; 2011-15 period; Data sources: The Web  of Science Core Collection, The Italian Ministry for Education, Universities and Research database (university staff information), and an archive of Italian economists’ media contributions; Cosine similarity; Logistic regression; Conditional effects of the Poisson model; N = 948 tenured academic economists. | Citations measure  versus scientific quality | - Italian academic economists’ citations cannot be unbiased proxies of scientific quality. |
| Dong et al. (2021) [45] | l 17 disciplines; The China’s basic research funding Program 973; A community life-cycle; A clique percolation method; The Pearson correlation coefficient; N = 177,909 articles. | Roles of top scientists in community development | - Top scientists can create stronger research communities, leading to faster progress and better results. |
| Fukugawa (2021) [4] | The METI USO Database; Elsevier’s SciVal; Logistic regression; N=620 university spinoffs in Japan. | The quality of science and university spinoffs | - Citation impacts are stronger than publication quality impacts on the market value of startups initialized by academic researchers. |
| Gorodnichenko  et al. (2021) [46] | The IDEAS/RePEc dataset; 2006–12 period; Regression analysis - linear model; N = 65,465 non-presented and 4,043 presented works from three major economic conferences in the U.K, Europe, and the U.S. | Differences in publication outcomes of presented and unpresented works | - Conference presentations improve publishing chances in high-quality journals for prominent and male authors. - Attending conferences can increase the chances of publishing in high-quality journals and improve metrics like citation count. |
| Kowaltowski  et al. (2021) [47] | Case study | Movement against “Quantophrenia” | - Proposal concerning the Initiative for Responsible Scientific Assessment to promote higher quality research assessment with its guidelines. |
| Liu et al. (2021) [48] | The Web of Science Core Collection; 1978–2017 period; Experiment; An indicator normalized relative level as an intermediate variable; PageRank algorithm; N = 852 journals of Mathematics, Physics, and Space Science, including 3,847,243 articles. | Paper impact calculation | - Three citation weighting functions and their comparison with PageRank. - The quality of citing items determines article ranking, not citation counts. |
| Morales et al. (2021) [9] | An online survey; September and October 2018; NVivo 12; the Kruskal Wallis test, chi square tests, Wilcoxon Rank sum test, and Spearman’s correlations for non-parametric data and one-way analysis of variance and Pearson’s correlations; N= 338 faculties (84 from Canadian institutions, 254 from the United States). | Defining the quality, prestige, and impact of academic journals | - Academic journal quality, prestige, and impact are defined in overlapping ways. - Diversity in definitions is not dependent on demographic characteristics. - Research evaluation reform is necessary. |
| Mryglod et al. (2021) [49] | The open Crossref data, Ukrainika Naukova database; The Scopus and the Web of Science databases; 2002–2020 period; A large-scale and longitudinal quantitative analysis;  N=123 Ukrainian journals in economics. | Specific characteristics of Ukrainian economic research | - Ukrainian economic research shows a trend toward more collaborative work. |
| Rowley  and Sbaffi (2021) [50] | Online survey; July - August 2019 period; One-way between-groups analysis of covariance; N=1,085 respondents. | Journal selection criteria | - Male and female scientists differ significantly in authority, discoverability, publication experience and confidence. |
| Wang et al. (2021) [51] | Qualitative study; Case of three Chinese publishers. | A Scientific publication system | - Chinese academic publishing is increasingly aligned with international standards, but the reviewing procedures of Chinese journals require improvement. |
| Aguinis et al. (2020) [13] | A qualitative study | The practice of counting A-journal publications in the field of management | - Valuing academic research has positive and negative consequences under the new bottom line: “an A is an A”. |
| Baas et al. (2020) [52] | Bibliometric approach; The Scopus database and Elsevier’s SciVal; Analysis of distributions; Precision and recall evaluated using several metrics. | Scopus as a high-quality source of bibliometric data | - Studies using the complete Scopus database or Elsevier’s SciVal have performed bibliometric analyses, including citation analysis and algorithm development, to enhance research evaluation. |
| Butler  and Spoelstra (2020) [53] | Qualitative study in the field of management; Semi-structured interviews; N = n.d. | Academic research as a “publication game’ | - Academic work can be play-mentality driven, and using the “publication game” metaphor can unintentionally affect academic behavior. |
| Sotomayor-Beltran (2020) [54] | Commentary; The case of Peruvian academic scientists. | Awareness of predatory publishing danger | - The call for Peruvian academics to produce high-quality articles and penalize publication in predatory journals. |
| Tonta 2020 [14] | The Web of Science database; 2006-15 period; Indexes: SCI-EXPANDED, SSCI, A&HCI; The stratified probability sampling technique; The Pearson correlation coefficient; The hurdle model used; The sample size for each year is close to 2% ; 1,679 TÜBİTAK-supported articles, and 31321 unsupported articles. | Impact of monetary support on citation counts | - Monetary support for academic scientists has little impact on citation counts. - Articles with and without support receive a similar number of citations per article. |
| Xie 2020 [1] | 1951-2018 period; Pearson correlation coefficient;  Auto-correlation coefficients; Poisson model: N = 220,344 pubs in 1586 journals and proceedings. | Publication productivity | - Proposal for a prediction model of the number of publications for groups of researchers using a piecewise Poisson. The model may not be appropriate for highly productive researchers. |

**Appendix 2. Demographic variables of survey respondents.**

| **Demographic variables** | | **Number  of respondents** | **Percentage  of respondents** | **Number  of respondents** | **Percentage  of respondents** | **Number  of respondents** | **Percentage  of respondents** |
| --- | --- | --- | --- | --- | --- | --- | --- |
| Gender |  | **Surveys 1 and 2 (Period 1)** | | **Surveys 1 and 2 (Period 2)** | | **Survey 1 (Periods 1 and 2)** | |
|  | Females | 238 | 50.0 | 229 | 50.4 | 62 | 40.8 |
|  | Males | 231 | 48.5 | 218 | 48.0 | 88 | 57.9 |
|  | Unspecified | 7 | 1.5 | 7 | 1.5 | 2 | 1.3 |
| Age | 20 - 34 | 86 | 18.1 | 81 | 17.8 | 22 | 14.4 |
|  | 35 - 49 | 228 | 47.9 | 220 | 48.5 | 71 | 46.7 |
|  | 50 - 68 | 146 | 30.7 | 138 | 30.4 | 51 | 33.6 |
|  | >69 | 16 | 3.4 | 15 | 3.3 | 8 | 5.3 |
| Position type | Graduate student in doctoral program | 52 | 10.9 | 50 | 11.0 | 13 | 8.6 |
|  | Lecturer | 53 | 11.1 | 51 | 11.2 | 18 | 11.8 |
|  | Assistant Professor | 143 | 30.0 | 137 | 30.2 | 45 | 29.6 |
|  | Associate Professor | 123 | 25.8 | 121 | 26.7 | 38 | 25.0 |
|  | Professor | 86 | 18.1 | 78 | 17.2 | 30 | 19.7 |
|  | Retired | 4 | 0.8 | 4 | 0.9 | 3 | 2.0 |
|  | Other: Assistant and researcher | 15 | 3.2 | 13 | 2.9 | 5 | 3.3 |
| Science  discipline | Social sciences | 382 | 80.3 | 364 | 80.2 | 119 | 78.3 |
|  | Engineering and technology | 65 | 13.7 | 63 | 13.9 | 24 | 15.8 |
|  | Humanities | 13 | 2.7 | 11 | 2.4 | - | - |
|  | Natural sciences | 14 | 2.9 | 14 | 3.1 | 9 | 5.9 |
|  | Medical and health sciences | 1 | 0.2 | 1 | 0.2 | - | - |
|  | Agricultural sciences | 1 | 0.2 | 1 | 0.2 | - | - |
| Teaching model | Traditional | 4 | 0.8 | 40 | 8.8 | 3 | 2.0 |
|  | Traditional and online | 26 | 5.4 | 395 | 87.0 | 6 | 3.9 |
|  | Online | 446 | 93.8 | 19 | 4.2 | 143 | 94.1 |
| Country | Poland | 278 | 58.4 | 266 | 58.6 | 102 | 67.1 |
|  | Abroad | 198 | 41.6 | 188 | 41.4 | 50 | 32.9 |

Types of positions may vary between countries. We assumed that lecturers as academics are mainly engaged in teaching, whereas professors are expected to conduct more research work.

Source: Authors’ computations based on [67].
